# Unraveling the microbiome of a thermophilic biogas plant by metagenome and metatranscriptome analysis complemented by characterization of bacterial and archaeal isolates

**DOI:** 10.1186/s13068-016-0581-3

**Published:** 2016-08-11

**Authors:** Irena Maus, Daniela E. Koeck, Katharina G. Cibis, Sarah Hahnke, Yong S. Kim, Thomas Langer, Jana Kreubel, Marcel Erhard, Andreas Bremges, Sandra Off, Yvonne Stolze, Sebastian Jaenicke, Alexander Goesmann, Alexander Sczyrba, Paul Scherer, Helmut König, Wolfgang H. Schwarz, Vladimir V. Zverlov, Wolfgang Liebl, Alfred Pühler, Andreas Schlüter, Michael Klocke

**Affiliations:** 1 Center for Biotechnology (CeBiTec), Institute for Genome Research and Systems Biology, Bielefeld University, Universitätsstr. 27, 33615 Bielefeld, Germany; 2 Department of Microbiology, Technische Universität München, Emil-Ramann-Str. 4, 85354 Freising-Weihenstephan, Germany; 3 Institute of Microbiology and Wine Research, Johannes Gutenberg-University, Becherweg 15, 55128 Mainz, Germany; 4 Dept. Bioengineering, Leibniz-Institut für Agrartechnik Potsdam-Bornim e.V. (ATB), Max-Eyth-Allee 100, 14469 Potsdam, Germany; 5 Faculty Life Sciences/Research Center ‚‘Biomass Utilization Hamburg’, University of Applied Sciences Hamburg (HAW), Ulmenliet 20, 21033 Hamburg-Bergedorf, Germany; 6 RIPAC-LABOR GmbH, Am Mühlenberg 11, 14476 Potsdam-Golm, Germany; 7 Faculty of Technology, Bielefeld University, Universitätsstr. 25, 33615 Bielefeld, Germany; 8 Department of Bioinformatics and Systems Biology, Justus-Liebig University Gießen, Heinrich-Buff-Ring 58, 35392 Giessen, Germany

**Keywords:** Anaerobic digestion, Biomethanation, Microbial community structure, Polyphasic characterization, Cellulolytic *Bacteria*, Acidogenic *Bacteria*, Acetogenic *Bacteria*, Methanogenic *Archaea*, Fragment recruitment, Culturomics

## Abstract

**Background:**

One of the most promising technologies to sustainably produce energy and to mitigate greenhouse gas emissions from combustion of fossil energy carriers is the anaerobic digestion and biomethanation of organic raw material and waste towards biogas by highly diverse microbial consortia. In this context, the microbial systems ecology of thermophilic industrial-scale biogas plants is poorly understood.

**Results:**

The microbial community structure of an exemplary thermophilic biogas plant was analyzed by a comprehensive approach comprising the analysis of the microbial metagenome and metatranscriptome complemented by the cultivation of hydrolytic and acido-/acetogenic *Bacteria* as well as methanogenic *Archaea*. Analysis of metagenome-derived 16S rRNA gene sequences revealed that the bacterial genera *Defluviitoga* (5.5 %), *Halocella* (3.5 %), *Clostridium* sensu stricto (1.9 %), *Clostridium* cluster III (1.5 %), and *Tepidimicrobium* (0.7 %) were most abundant. Among the *Archaea*, *Methanoculleus* (2.8 %) and *Methanothermobacter* (0.8 %) were predominant. As revealed by a metatranscriptomic 16S rRNA analysis, *Defluviitoga* (9.2 %), *Clostridium* cluster III (4.8 %), and *Tepidanaerobacter* (1.1 %) as well as *Methanoculleus* (5.7 %) mainly contributed to these sequence tags indicating their metabolic activity, whereas *Hallocella* (1.8 %), *Tepidimicrobium* (0.5 %), and *Methanothermobacter* (<0.1 %) were transcriptionally less active. By applying 11 different cultivation strategies, 52 taxonomically different microbial isolates representing the classes *Clostridia*, *Bacilli*, *Thermotogae*, *Methanomicrobia* and *Methanobacteria* were obtained. Genome analyses of isolates support the finding that, besides *Clostridiumthermocellum* and *Clostridium stercorarium,Defluviitoga tunisiensis* participated in the hydrolysis of hemicellulose producing ethanol, acetate, and H_2_/CO_2_. The latter three metabolites are substrates for hydrogentrophic and acetoclastic archaeal methanogenesis.

**Conclusions:**

Obtained results showed that high abundance of microorganisms as deduced from metagenome analysis does not necessarily indicate high transcriptional or metabolic activity, and vice versa. Additionally, it appeared that the microbiome of the investigated thermophilic biogas plant comprised a huge number of up to now unknown and insufficiently characterized species.

**Electronic supplementary material:**

The online version of this article (doi:10.1186/s13068-016-0581-3) contains supplementary material, which is available to authorized users.

## Background

As consequence of the Kyoto Protocol, approved in 1997, and the therein specified urgent demand regarding reduction of greenhouse gas emissions, the policy for energy transition was intensified in Germany [1]. This strategy essentially implies utilization of renewable biomass for the generation of heat, electricity and fuels. In Germany approximately 8000 biogas plants are currently generating permanently more than 3.8 GW electric power and 1 GW heat energy for more than nine million households. Substrates are renewable resources such as so-called ‘energy’ crop silage, manure and sludge from animal husbandry, and organic residues from industry and agriculture [2, 3]. The energy production from biogas avoids the emission of approximately 16.8 million tons of the climate-relevant gas carbon dioxide (CO_2_) [2]. In addition, bio-methane production from 'energy crops' and plant residues is the most efficient bioenergy production pathway [4].

Despite the ecological and economical importance of biogas generation, the microbial networks responsible for anaerobic digestion and biomethanation of biomass are still poorly understood. The classical concept of understanding the biogas process from biomass to biogas is assuming a more or less linear degradation pathway beginning with the hydrolytic breakdown of complex biomass compounds towards short-chained volatile fatty acids (VFA) (acidogenesis), which are subsequently converted mainly to acetic acid (acetogenesis) and gases, mainly CO_2_ and molecular hydrogen (H_2_). Acetic acid is regarded as one of the major precursors of methane (CH_4_, acetoclastic methanogenesis), and for a long time was considered to be the primary origin of methane (up to 73 %) [5].

An alternative pathway for methanogenesis is the H_2_-mediated reduction of CO_2_, released during acidogenesis or as product of the oxidation of acetate (hydrogenotrophic methanogenesis). Several studies on the microbial community structure within biogas plants revealed the importance of this long-time neglected pathway for large-scale biogas production, especially when carbohydrate substrates are abundant, at stressed conditions, namely at high ammonia concentrations or short hydraulic retention times, and/or during anaerobic digestion of crop material [6, 7].

Furthermore, advanced molecular analysis, such as high-throughput DNA sequencing of microbial 16S rRNA genes and microbial metagenomes derived from biogas plants, showed that the microbial community architecture is much more complex and may include up to several hundreds or even thousands of microbial species [8, 9]. As an example, members of the phylum *Firmicutes*, namely several species of the genus *Clostridium* such as *C. thermocellum* or *C. stercorarium*, were commonly assumed to represent the major degraders of complex plant carbohydrates, such as cellulose and hemicelluloses, especially xylanes, and be the primary producers of VFA, acetic acid, and CO_2_/H_2_ [10]. However, molecular studies pointed also to the participation of members of other phyla in the anaerobic degradation process, namely those of the phyla *Chloroflexi*, *Proteobacteria*, *Synergistetes*, and, predominantly, *Bacteroidetes* [11–15].

Both, microbiological and molecular studies for characterization of biogas communities were mostly applied on anaerobic digesters operated at mesophilic temperatures. As an example, a survey conducted in 2005 of in total 413 randomly chosen biogas plants revealed that approximately 86 % of the biogas plants in Germany are operated at mesophilic conditions with temperatures ranging from 37 to 43 °C [16]. Only few plants, i.e. 6 %, performed the biomethanation under thermophilic temperature regime; 4 % are staged reactors combining thermophilic and mesophilic fermenters.

Thermophilic plants have the reputation to be less stable than mesophilic ones. However, a number of studies revealed the advantages of thermophilic digestion, namely a faster hydrolysis and acidogenesis even at increased ammonia concentrations combined with a higher methane yield as well as a shorter hydraulic retention time of the biomass (about 20 days compared to about 70 days in mesophilic biogas plants) with the additional benefit of hygenization of the input material [17, 18]. Due to the limited number of thermophilic biogas plants, studies on the associated microbial trophic networks are still limited and mostly focused on waste, wastewater or manure digesting plants [18]. Hence, thermophilic microbial consortia appear to be less well understood than mesophilic ones.

Despite the undoubted advances in microbial ecology by the introduction of microbial metagenomics, -transcriptomics, and -proteomics, a major drawback of all these approaches is the huge number of un-assignable sequences [15, 19, 20]. This is due to the still highly limited availability of reference strains and their corresponding genomes in public databases. Consequently, for a detailed characterization of complex microbial consortia, commonly a polyphasic approach is recommended involving parallel application of both, traditional cultivation as well as molecular analyses.

In this study, for the first time, such a comprehensive polyphasic approach was applied to unravel the structure and the functionality of the microbial consortium within an industrial-scale thermophilic biogas plant optimized for anaerobic digestion and biomethanation of 'energy crops'. In this plant, maize and barley silage were anaerobically digested together with cattle and pig manure at a thermophilic temperature regime (54 °C). The polyphasic analysis included (i) characterization of the microbial community structure by high-throughput metagenomic 16S rRNA gene sequencing; (ii) determination and analysis of metabolically active microorganisms by high-throughput metatranscriptomic 16S rRNA tag sequencing; (iii) functional community profiling by metagenome sequencing and analysis; (iv) identification and metabolic characterization of isolates for cellulolytic/hydrolytic, acidogenic/acetogenic, and methanogenic microbial species, and (v) characterization of the genetic potential of the isolates by genome sequencing and analysis. The overall aim was the compilation of the core microbiome and its functional characterization for a thermophilic biogas plant.

## Methods

### Sampling of an industrial-scale thermophilic biogas plant

**Fig. 1:**
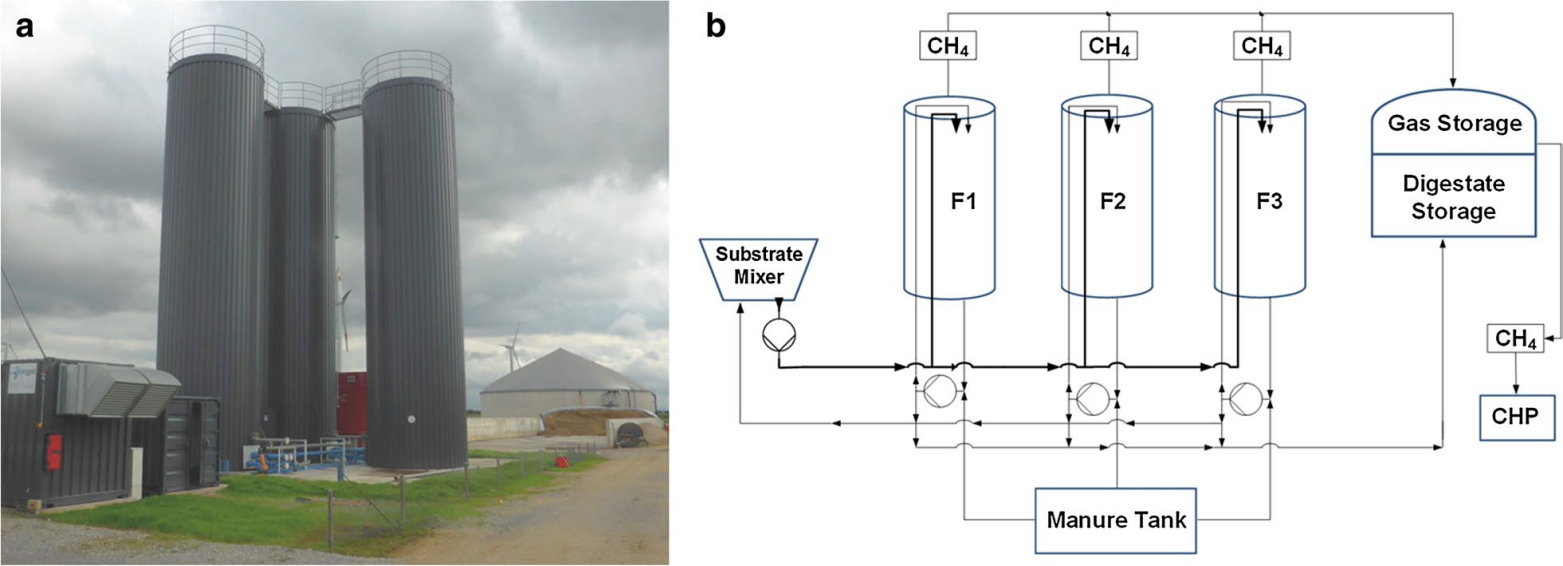
Picture (**a**) and flow chart (**b**) of the sampled thermophilic biogas plant. **a** *Left side* central heating and power plant; *middle* the three main fermenters; *right side* unheated effluent tank

The thermophilic biogas plant (54 °C) analyzed is located in Viersen (North Rhine-Westphalia, Germany) and is part of an agricultural pig farm with 800 sows and about 24,000 piglets per year. The biogas plant consists of three connected identical cylindrical digesters (height 14 m, diameter 3.3 m, operating volume 105 m^3^) operated in parallel (Fig. 1) and fed with similar substrate mix. After the fermentation, the digestate is stored in an unheated digestate tank (operation volume 2500 m^3^) for at least 240 days. At time point of sampling, the following substrates were used for biomethanation: maize silage (56 % fresh mass, FM), barley (6 % FM), cattle manure (6 % FM), and pig manure (32 % FM). In total, every fermenter received 5.3 t_FM_ d^−1^. The total dry matter (DM) content of the fermenters ranged between 10 and 12 %, about equal to a volatile solid (VS) content between 8–9 %. Feeding of substrates followed eight times per day. Trace elements, i.e. Fe, Mn, Zn, Cu, Mo, Co, Ni, and Se were permanently supplemented in rates below 1 ppm. Mixing of fed substrates occurred continuously in the fermenters (24 h d^−1^) by an eccentric screw pump. The hydraulic retention time (HRT) of solid substrates in the fermenters F1–F3 was 19.8 d and high organic loading rate (OLR) of 8 kg _VS_ m^−3^ d^−1^. The biogas plant produced permanently and at the same time 175 kW electric energy and approximately 190 kW thermic energy by a central heating and power plant.

Samples of 0.5–1.0 l fermentation sludge were collected from digesters F1 and F2 (Fig. 1). For DNA and RNA extraction, fermentation samples were processed immediately after transport to the laboratory. For cultivation experiments samples were stored overnight at 4 °C; for all further applications samples were stored at −18 °C until usage.

### Chemical analysis

All samples were centrifuged and the supernatant was acidified by addition of ortho-phosphoric acid to obtain a final pH value of 2.0. Total VFA, i.e. C_1_–C_6_ short chain fatty acids, alcohols, lactic acid, and phenyl acetic acid, were determined gaschromatographically using an Agilent HP 5890 Series II gas chromatography system equipped with a flame ionization detector (FID) and a BP 21 bonded FFAP fused silica column (length 250 mm, diameter 0.53 mm, thickness of the immobilized phase 0.5 µm) [21]. The FID was used in the automatic and splitless mode. An automatic sampler injected 1 µl sample under the temperature program of 240/70–235/260 °C for injector, column and detector, respectively. H_2_ was applied as carrier gas with a H_2_-flow of 30 ml min^−1^. The air-flow of the FID was 300 ml min^−1^ with a makeup-flow of 25 ml nitrogen per min. The total run time was adjusted to 30 min. A weekly calibration was performed with a commercial external standard (Supelco 46975-U, Sigma-Aldrich, Germany). DM and VS content were determined according to the standard guidelines, i.e. VDI 4630 protocols [22].

### Microscopic analysis

Microscopic determinations of cell morphologies and titers were performed with a DM6000B fluorescence microscope (Leica, Germany) fitted with a motorized and PC-controlled three-axis cross table. Images were captured by a DFC365FX camera (Leica), controlled and analyzed with the Image Pro 7 software (MediaCybernetics, USA). For each sample, approximately 20 images were captured in succession in the chosen area at 400-fold magnification. For detection of methanogens, the fluorescence filter set CFP (Leica), excitation 426–446 nm, emission 460–500 nm was used. A second filter set L5 (Leica) with an excitation of 460–500 nm and an emission of 512–542 nm was used for counting total cells stained with SYBR Green I. Shutter speed was 82.4 ms; and signal intensification of the picture was four times. Both values were kept for all experiments. Further details on the procedure were published previously [23].

### Extraction of total microbial DNA, metagenome library preparation and sequencing

Total microbial DNA was extracted applying four different commercial DNA preparation kits (protocols A–D) and the CTAB-based DNA extraction protocol (protocol E; details as published by [8]) in parallel: (protocol A) FastDNA spin kit for soil (MP Biomedicals, France), using ceramic and silica particles and a lysing matrix for cell fracturing; (protocol B) NucleoSpin Soil kit (Macherey–Nagel, Germany) based on ceramic beads in lysis buffer; (protocol C) PowerLyzer DNA isolation kit (MoBio, USA) based on a glass bead solution; (protocol D) PowerSoil DNA isolation kit (MoBio, USA), using a glass bead solution; (protocol E) chloroform-isoamyl alcohol DNA extraction without a bead-beating step.

For bead-beating (protocols A–D), the kit-specific columns were transferred to a FastPrep FP 120 Ribolyzer (Thermo Savant, Germany) and agitated at level six for 1 min. Further steps were performed in accordance with manufacturer’s guidelines.

For whole metagenome library sequencing of the five DNA extracts, 1 µg DNA of each sample was used. Genomic DNA fragmentation into approx. 550 bp fragments was carried out applying the Rapid Library Nebulizer kit and the GS Rapid Library Nebulizer device (Roche, Germany). Finally, the Illumina TruSeq^®^ DNA PCR-free sample preparation kit (Illumina, USA) was used to construct five sequencing libraries, which were sequenced on an Illumina MiSeq system applying the paired-end protocol and utilizing the V3 kit chemistry with 600 cycles (2 × 300 bp).

### Extraction of total microbial RNA, metatranscriptome library preparation and sequencing

In order to extract the total microbial RNA, the modified protocol published previously [19] was applied. The modifications are described in the Additional file 1. The mRNA library was prepared from samples with the appropriate quality and quantity using the TrueSeq Stranded mRNA Sample Prep kit (Illumina, USA). Sequencing was performed using the Illumina V2 chemistry (2 × 250 bp) and applying the MiSeq paired-end mode.

### Metagenome and metatranscriptome sequence data analysis

Obtained metagenomic raw sequences were quality filtered and separated by the different multiplex identifiers. In total, five metagenome datasets were obtained, one for each DNA extraction method. Overlapping paired-end reads from each dataset were merged together applying the computational tool Flash [24]. 1.3 million randomly extracted fragments from each dataset were combined to a total of 6.5 million sequences and imported into the metagenome annotation platform MGX [25] for taxonomic and functional analysis. Finally, all metagenome reads were compared with the ribosomal database project (RDP) database [26] in order to identify encoded 16S rRNA genes in the metagenome. Therefore, the ‘16S pipeline’ implemented in MGX was used.

Due to the lack of *Defluviitoga* 16S rRNA gene sequences in the RDP database at the point of analysis, additional investigation of *Thermotogae* sequences from the analyzed biogas plant was performed. Therefore, the 16S rRNA gene sequence of *Defluviitoga tunisiensis* str. L3 [27] was compared against the 16S rRNA gene amplicon sequences applying BLASTN analysis with 97 % sequence identity and *e* value of 1e^−10^.

Raw sequencing data are available in the EBA database under study accession numbers PRJEB12913 (for the metagenome dataset) and PRJEB12916 (for the metatranscriptome dataset).

To characterize the gene content of the microbial community, all reads were functionally annotated using the clusters of orthologous groups of proteins database (COG) [28, 29] with a BLASTX search of reads vs the COG database applying MGX pipeline defaults. The occurrence of the carbohydrate-active enzymes was predicted using the carbohydrate-active enzyme database annotation web-server dbCAN [30] also applying MGX standard settings.

For taxonomic characterization of the metabolically active biogas community the obtained metatranscriptome sequences were quality filtered and uploaded into MGX. The RDP database was used to identify all 16S rRNA gene transcripts obtained from the sequenced metatranscriptome for taxonomic profiling. Here, the ‘16S pipeline’ from MGX was used.

### Cultivation, isolation, and culture-based characterization of microorganisms

For cultivation and culture-based identification of members of the microbial community within the main digesters of the biogas plant, in total 11 different isolation strategies were applied with respect to the different trophic microbial groups involved in anaerobic digestion of biomass and subsequent biomethanation. Exemplary work-flow pathways are depicted in the Additional file 1: Figure S1. Details on the isolation strategies are provided as Additional file 1. Briefly, following protocols for isolation of fermentative *Bacteria* and for isolation of methanogenic *Archaea* were used.

*Isolation strategy (1) targeting mesophilic pathogenic* Bacteria: Serial dilutions were plated on Columbia Blood Agar, Columbia Blood Agar with Neomycin, Columbia Blood Agar with Gentamycin, Plate Count Agar, Polymyxin Egg Yolk Mannitol Bromothymol Blue Agar, and Sabouraud Dextrose Agar with Chloramphenicol and incubated under microoxic and anoxic conditions at 37 °C for 48 h. The anaerobic culture conditions were generated using the AnaeroGen 2.5 l (Atmosphere Generation System, Thermo Scientific, Oxoid Basingstoke, UK). All colonies with similar morphology were counted and identified using the MALDI-TOF MS method. For this purpose, the MALDI-TOF MS sample preparation was performed as described previously [31]. Further details are given below.

*Isolation strategy (2) targeting thermophilic pathogenic* Bacteria: Similar to strategy (1), but with cultivation at 50 °C.

*Isolation strategy (3) targeting cellulolytic* Bacteria: GS2 medium [32] or mineral medium [33] supplemented with 0.5 % (w/v) Avicel in liquid cultures and 0.5 % (w/v) phosphoric acid swollen cellulose (PASC) [34] or Avicel on solid agar plates together with cattle rumen content or digestate from a biogas plant were used to enrich for cellulolytic bacteria under anoxic conditions at 55 °C.

*Isolation strategy (4) targeting cellulolytic* Bacteria: Similar to [35], with some modifications as described in detail in the Additional file 1.

*Isolation strategy (5) targeting cellulolytic* Bacteria: Similar to strategy (3), with the following modifications: the dilution of the suspended sludge was directly plated on agar plates containing 0.05 % (w/v) of cellobiose as carbon source and overlaid with GS2 Agar (2 % w/v) containing 0.5 % (w/v) Avicel or PASC.

*Isolation strategy (6) targeting acidogenic/acetogenic* Bacteria: Modified minimal DSMZ medium 287 [36] supplemented with one carbon source (6 g l^−1^ of Na^+^-DL-lactate, succinate, glucose or a mixture of the following six amino acids: l-alanine, l-serine, l-threonine, L-cystein, l-glutamic acid, and l-methionine) was used for cultivation under anoxic conditions at 54 °C. To obtain pure cultures, the deep agar shake method was applied [37].

*Isolation strategy (7) targeting acidogenic/acetogenic* Bacteria: Similar to strategy (6), with the following modification: instead of the deep agar shake method, plating on anoxic agar medium was performed using the above-mentioned nutrition media [37].

*Isolation strategy (8) targeting different anaerobic thermophilic* Bacteria: To isolate a variety of different bacteria, media with different substrates were used: (a) R2A medium [38]; (b) nitrate broth medium [39]; (c) minimal medium [38] supplemented with formate and glucose; (d) BM/NO^3−^ medium [40]; (e) DSMZ medium 287 [36] supplemented with acetate, formate, and methanol [41]. Aliquots of serial dilutions were spread on pre-reduced agar plates of the respective medium and incubated under anoxic conditions at 50 °C.

*Isolation strategy (9) targeting facultative anaerobic thermophilic* Bacteria: The reactor sample was diluted 10^4^- and 10^6^-fold, plated on 10 % DEV nutrient agar (Merck, Germany) and incubated under exposure to air oxygen at 50 °C. Single colonies were picked and re-streaked for purification.

*Isolation strategy (10) targeting methanogenic* Archaea: A cultivation technique for strictly anaerobic microorganisms was performed in accordance to the recommendations by [42]. Details on the nutrient media and the applied antibiotics are provided as Additional file 1.

*Isolation strategy (11) targeting methanogenic* Archaea: As described by [42], colonies picked from deep agar shake (medium DSMZ 287 supplemented with an amino acid solution) were incubated at 55 °C and then moved to the selective cultivating temperature, e.g. 65 °C. A combination of the antibiotics ampicillin and vancomycin was applied.

### Identification, abundance determination and phylogenetic allocation of isolates

For the identification of the bacteria isolated with strategies (1) and (2), the MALDI-TOF MS analysis was performed [31]. Therefore, each colony with different morphology was analyzed. A small amount of cell colony (~10^5^–10^7^ cells) was transferred from an agar plate directly on the MALDI-target and mixed with alphacyno-4-hydroxy cinnamic acid (CHAC, saturated solution in 33 % acetonitrile, 33 % ethanol, 3 % formic acid) matrix solution. The mass spectra were acquired with an AXIMA Confidence MALDI-TOF MS (Shimadzu Europe) in a mass range *m*/*z* from 3000 to 20,000. The Shimadzu Biotech Launchpad software (Shimadzu Europe) was used for spectra acquisition and peak detection. All mass spectra were identified with the SARAMIS software (database V 3.10, VitekMS Plus; bioMerieux, Nürtingen, Germany). The commercial data base was updated with ~20,000 reference spectra of isolates of different origin including biogas samples over the last 7 years. All settings and guidelines from the manufacturer for the standard application were used.

*Bacteria* and *Archaea* obtained with the isolation strategies (3)–(11) were identified by 16S rRNA gene sequence analysis using the EzTaxon identification tool [17, 43]. Details are provided as Additional file 1.

To quantify the abundance of the isolated strains within the thermophilic microbial community, combined metagenome and metatranscriptome sequences were mapped against the 16S rRNA gene sequence of the obtained isolates applying the gsMapper 2.8 program (Roche, Germany). For this purpose, BLASTN analysis with a minimum sequence identity of 97 % and a minimal sequence overlap of 90 % was used to align the sequencing reads to the isolates’s 16S rRNA gene sequences.

To determine the phylogenetic relationship between the different isolates and the corresponding closest related type strains, a phylogenetic tree was constructed based on 16S rRNA gene sequences applying the ARB program [44]. Therefore, 16S rRNA genes of the isolates were amplified (approximately 1100 nt in length) and sequenced as described in the Additional file 1. The 16S rRNA gene sequence of the related type strains was obtained from the Silva ribosomal RNA project [45]. All sequences were aligned using the high-throughput multiple sequence alignment tool SINA [46]. Subsequently, the resulting multiple sequence alignment was introduced into the phylogenetic tree containing selected 16S rRNA gene sequences of previously described bacterial and archaeal type strains as provided by the SILVA database [47]. To detect phylogenetically different isolates, the 16S rRNA gene sequences of closely related isolates were compared to each other using the ARB distance matrix tool. Isolates were considered to be phylogenetically different when their 16S rRNA gene sequences showed less than 100 % identity.

### Genome sequencing, annotation and analysis of reference strains

For genome sequencing and analysis, three bacterial strains originating from the analyzed biogas plant, namely *Clostridium* resp. *Ruminiclostridium cellulosi* str. DG5 (taxonomic denomination under revision) [48], *Herbinix hemicellulosilytica* str. T3/55^T^ [49] and *Defluviitoga tunisiensis* str. L3 [27, 37] were selected. The isolation of genomic DNA was accomplished applying the DNeasy Blood and Tissue Kit (Qiagen, Hilden, Germany) in case of *D. tunisiensis* str. L3 and classic chloroform/isoamyl alcohol DNA extraction for *C. cellulosi* str. DG5 and *H. hemicellulosilytica* str. T3/55^T^. Furthermore, 4 µg of total DNA were used to construct two 8-kb mate-pair sequencing libraries (Nextera Mate Pair Sample Preparation Kit, Illumina Inc.) and sequenced on an Illumina MiSeq system, applying the paired-end protocol. Obtained sequences were de novo assembled using the GS de novo Assembler software (version 2.8, Roche). Finally, an in silico gap closure approach was performed [50]. Gene prediction, annotation and pathway reconstruction of the sequenced genomes were accomplished using the GenDB platform [51]. To predict genes encoding carbohydrate-active enzymes the carbohydrate-active enzyme database (CAZy) annotation web-server dbCAN was used [30].

### Fragment recruitment

In order to determine species within the biogas plant affiliated to the strains *C. cellulosi* str. DG5, *H. hemicellulosilytica* str. T3/55^T^ and *D. tunisiensis* str. L3, the corresponding metagenome sequences were mapped on these three genomes as described recently [52]. Therefore, the combined metagenome sequencing dataset (see above) was used, representing the thermophilic biogas-producing microbial community. Afterwards, the FR-HIT software tool [53] was used to perform a global alignment against the completely sequenced genomes of the strains described above. An identity cutoff of 75 % was used to align the sequencing reads to the corresponding genome. Finally, the fragment recruitment was visualized by plotting the identity of the alignment against the alignment position on the corresponding genome sequence.

## Results

### Properties of the produced biogas and the digester liquid

To investigate the process performance of the biogas plant analyzed at the sampling time point, fermentation samples were analyzed regarding their physico-chemical characteristics. The stable anaerobic digestion performance was indicated by a low level of total VFA between 500 and 1000 mg l^−1^ [54]. At the day of sampling, the methane content of the biogas was 54 % (v/v); the CO_2_ content was 36 % (v/v). The daily gas production was on average 730 m^3^ per digester; and the daily electricity power production lay between 57 and 60 kWh per digester. Gas production was calculated from the produced electricity of the central heating power plant assuming a conversion efficiency of 37 %. The process fluid of the sampled digester, from which the microbiological analysis was performed, had the following physico-chemical characteristics: pH value 8.2; conductivity 22.2 mS cm^−1^ equalizing to 13.2 g KCl l^−1^, alkalinity 12,910 mg CaCO_3_ l^−1^, total VS 7.2 %, total DM content 9.7 %, acetic acid concentration (conc.) 908 mg l^−1^, propionic acid conc. 83.4 mg l^−1^, iso-butyric acid conc. 10.8 mg l^−1^, butyric acid conc. 5.3 mg l^−1^, iso-valeric acid conc. 10.6 mg l^−1^, valeric acid conc. 2.7 mg l^−1^, NH_3_ conc. 1056 mg l^−1^, NH_4_^+^ conc. 2873 mg l^−1^, PO_4_^3−^ conc. 298 mg l^−1^. These findings correlate with the parameters of agricultural biogas plants previously described by Laaber et al. [55].

### Microscopic determination of microbial cell number and shape

**Fig. 2:**
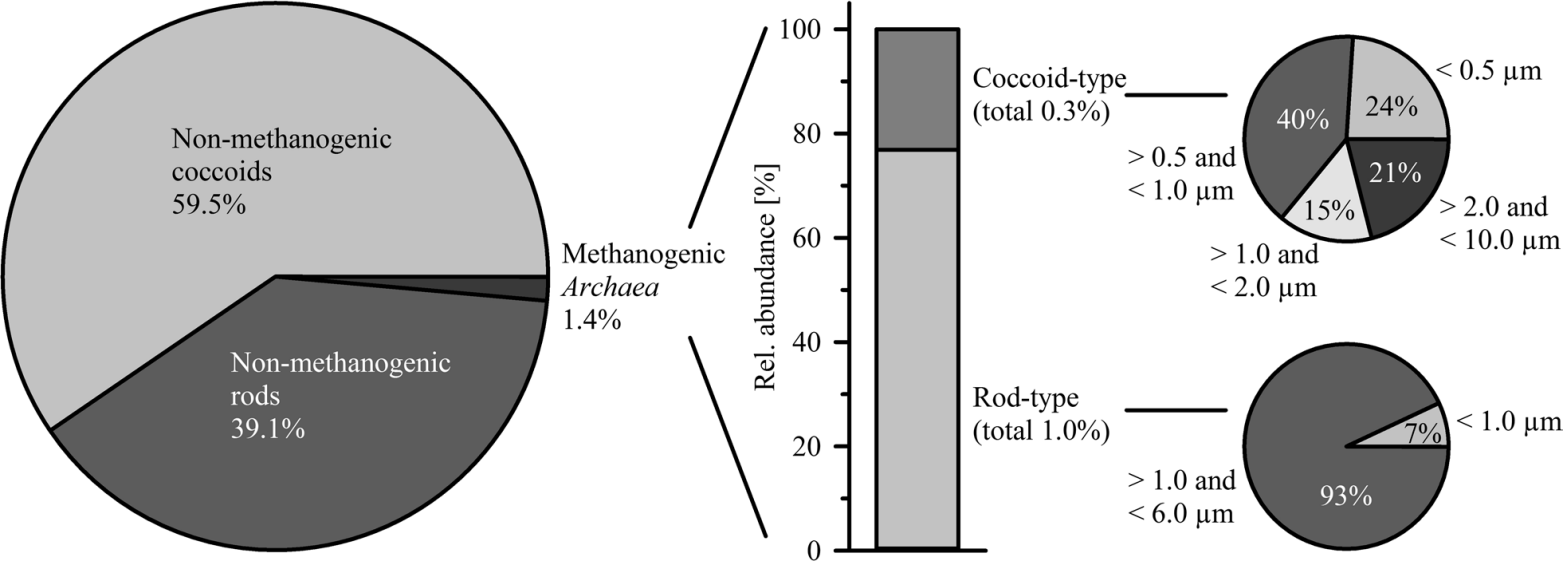
Morphological classification of microorganisms present in the thermophilic biogas reactor sample. For morphological classification, microscopy combined with image analysis was applied

The biogas reactor sample revealed 3.8 × 10^10^ total cells per ml process liquid (± 16 % standard deviation, SD) as determined by the quantitative microscopic fingerprinting (QMF) technique [23]. Methanogenic *Archaea* were determined with only 5.1 × 10^8^ cells per ml (±20 % SD), i.e. 1.4 % of the total cell number (Fig. 2).

Non-methanogenic *Bacteria* accounted for 98.6 % of the total cell counts, mostly cocci and rod-shaped cells. Long non-methanogenic rods (>6 µm) were not observed. The majority of the methanogenic *Archaea* were rod-shaped with a length of up to 6.0 µm (Additional file 1: Figure S5), i.e. about 80 % of the methanogens. Methanogenic rods longer than 6.0 µm were not found. Coccoid-type methanogens were present in lower amounts than rod-type methanogens, i.e. about 20 % of the methanogens. Their diameters ranged from values below 0.5 µm to above 2 µm (Fig. 2).

### Analysis of the microbial community structure based on 16S rRNA gene sequences extracted from metagenome sequence data

To determine the microbial community composition of the thermophilic biogas plant, high-throughput whole microbial metagenome sequencing was conducted. To cover the microbial diversity of the biogas plant as completely as possible, five different DNA extraction methods were applied resulting in generation of five metagenome datasets comprising between 2,803,170 and 5,975,856 sequences (Additional file 1: Table S1), which were finally combined into one equilibrated single dataset. From this combined metagenome dataset containing in total 6.5 × 10^6^ sequences, 16S rRNA gene sequences were extracted to determine the taxonomic profile of the thermophilic biogas microbiome by means of the RDP classifier implemented in MGX.

Out of in total 21,888 16S rRNA gene sequences, 18,817 sequences of prokaryotic origin (i.e. 85.9 % from all 16S rRNA gene sequences) were classified; 3071 sequences (14.0 %) remained with no further taxonomic assignment (Additional file 1: Table S2). For further analysis, the 18,817 16S rRNA gene sequences assigned to either the domain *Bacteria* or *Archaea* were taken as 100 %.

The thermophilic biogas community was dominated by *Bacteria* (94.5 % of all classified 16S rRNA gene sequences), with the remaining 5.4 % being classified as *Archaea*. Within the bacterial domain, high percentages could be assigned to the phyla *Firmicutes* (36.5 %), *Thermotogae* (7.1 %), and *Bacteroidetes* (4.5 %). The classes *Clostridia* (22.4 %), *Thermotogae* (7.1 %) and *Negativicutes* (0.8 %) belonging to the above listed phyla represented the major bacterial classes in the analyzed thermophilic biogas fermenter.

**Fig. 3:**
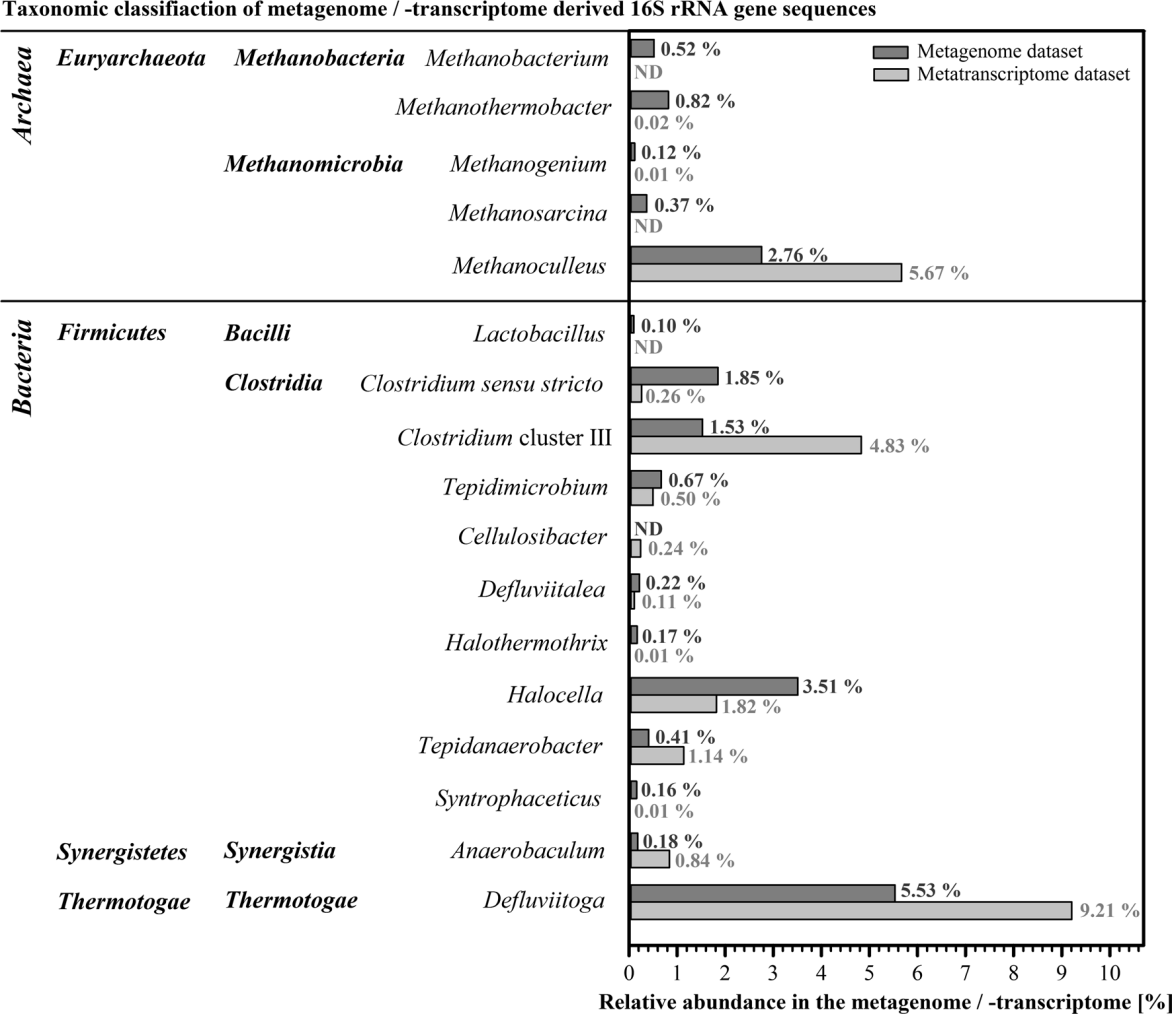
Relative abundance of the most abundant genera present in the analyzed thermophilic biogas plant. Abundances were determined based on 16S rRNA gene sequences derived from the metagenome (*dark grey bars*) or the corresponding metatranscriptome dataset (*light grey bars*) (≥0.1 % of sequences). The number of metatranscriptome-derived sequences, i.e. 532,381, was normalized to the number of metagenome-derived sequences, i.e. 18,817. *ND* not detected

Among the bacterial families, *Petrotogaceae* (6.4 %) (phylum *Thermotogae*) as well as *Halaerobiaceae* (4.8 %), *Clostridiaceae* cluster I (3.0 %), and *Ruminococcaceae* (2.7 %) (all phylum *Firmicutes*) occurred most frequently (Additional file 1: Table S3). The most prominent genera within the bacterial community were *Defluviitoga* (5.5 %) followed by *Halocella* (3.5 %), *Clostridium* sensu stricto (1.9 %), *Clostridium* cluster III (1.5 %), and *Tepidimicrobium* (0.7 %). (Fig. 3; Additional file 1: Table S3).

However, it should be noted that only 17.5 % of the identified 16S rRNA gene sequences could be assigned to known genera. Even if the applied approach is not appropriate to provide 16S rRNA gene sequences of sufficient length for species identification, the obtained results strongly indicate that the majority of the microbial genera or even species existing in this biogas plant, and presumably participating in anaerobic digestion, are still unknown (Additional file 1: Table S2).

Within the domain *Archaea*, exclusively members of the phylum *Euryarchaeota* were detected. The orders *Methanomicrobiales* (3.5 %) and *Methanobacteriales* (1.3 %) were the predominating taxa, with *Methanoculleus* (2.8 %) and *Methanothermobacter* (0.8 %) being the dominant genera (Fig. 3; Additional file 1: Table S3; Figures S2, S3).

To evaluate the diversity of the studied microbial community, the Shannon index was computed based on 16S rRNA gene fragments classified on the genus rank with at least 80 % confidence. This approach has been applied in ecological studies as an estimate of biodiversity, accounting for the number of different taxa as well as their relative abundance [11]. For the monitored thermophilic biogas plant, a Shannon index value of 2.55 was computed, which is slightly lower compared to values previously reported in studies analyzing mesophilic biogas digesters [56, 57]. However, limited biodiversity under thermophilic conditions is a commonly observed phenomenon [58, 59].

### Characterization of the metabolically active part of the microbial community based on 16S rRNA tags within a metatranscriptome dataset

To identify the metabolically active microbial community members, high-throughput whole metatranscriptome sequencing was performed. For this purpose, total microbial RNA was extracted, converted to cDNA and sequenced without prior removal of ribosomal RNA as published previously [60]. Sequencing on the Illumina MiSeq system resulted in 1,149,525 reads. 547,373 reads (47.6 %) were classified as encoding 16S rRNAs, whereas the remaining reads mainly arose from 5S rRNA and 23S rRNA genes; reads derived from mRNA and other RNA species were identified in minor amounts. From 532,283 taxonomically classified 16S rRNA sequences, 494,200 sequences were assigned to *Bacteria* (92.8 %) and 3181 were of archaeal origin (7.1 %). Altogether, 134,563 16S rRNA sequences (25.2 %) were identified at the genus level indicating that the majority of metabolically active microorganisms in the thermophilic biogas community is unknown as also indicated by the DNA-based analysis (Additional file 1: Table S2).

The taxonomic profile based on metatranscriptome 16S rRNA tags was compared to the corresponding profile deduced from metagenome sequences to determine metabolically active taxa within the community. Figure 3 and Additional file 1: Table S3 show the percentage of the most abundant genera as classified from both datasets. Surprisingly, the genus *Defluviitoga* (phylum *Thermotogae*) was most dominant in both datasets. Moreover, its fraction was higher within the metatranscriptome (9.2 %) than within the metagenome (5.5 %), indicating that members of this taxon were more active in the biogas plant in relation to their abundance. This was also true for the second and third most abundant genera within the metatranscriptome, *Methanoculleus* (phylum *Euryarchaeota*) and *Clostridium* cluster III (phylum *Firmicutes*), which showed a percentage of 2.8 and 1.5 % in the metagenome, but 5.7 and 4.8 % in the metatranscriptome, respectively. A similar relationship between metagenomic and metatranscriptomic assignments was also observed for the genera *Tepidanaerobacter* (phylum *Firmicutes*), *Anaerobaculum* (phylum *Synergistetes*), and *Cellulosibacter* (phylum *Firmicutes*), however, all featuring percentages below 3 %.

In contrast, the relative abundance of the genus *Halocella* (phylum *Firmicutes*) is higher within the metagenome (3.5 %) than in the metatranscriptome (1.8 %). This observation indicates that its metabolic activity was lower in relation to its abundance in the analyzed thermophilic biogas plant. Similar results were obtained for 17 more genera, all featuring percentages of less than 3 % in the metagenome as well as in the metatranscriptome datasets.

### Genetic potential of the microbial community determined by metagenome analysis

The conversion of organic material to biogas is a complex process involving a series of biochemical reactions starting with the degradation of mainly carbohydrate compounds, i.e. polysaccharides, especially cellulose, hemicellulose, starch, pectins, etc., but also proteins and lipids from plant material into less complex oligomeric and monomeric compounds such as oligosaccharides, monosaccharides, amino acids, and fatty acids. These are then further metabolized to fermentation end-products such as CO_2_, H_2_, acetate, and/or methylamines (from e.g. choline), which are precursors for methane production by methanogenic *Archaea*. In addition, NH_4_^+^ resulting from anaerobic degradation of amino acids is a nitrogen source for the methanogens. To determine the metabolic potential of the analyzed biogas microbiome, functional annotation of the obtained metagenome dataset was carried out applying the metagenome analysis platform MGX.

**Fig. 4:**
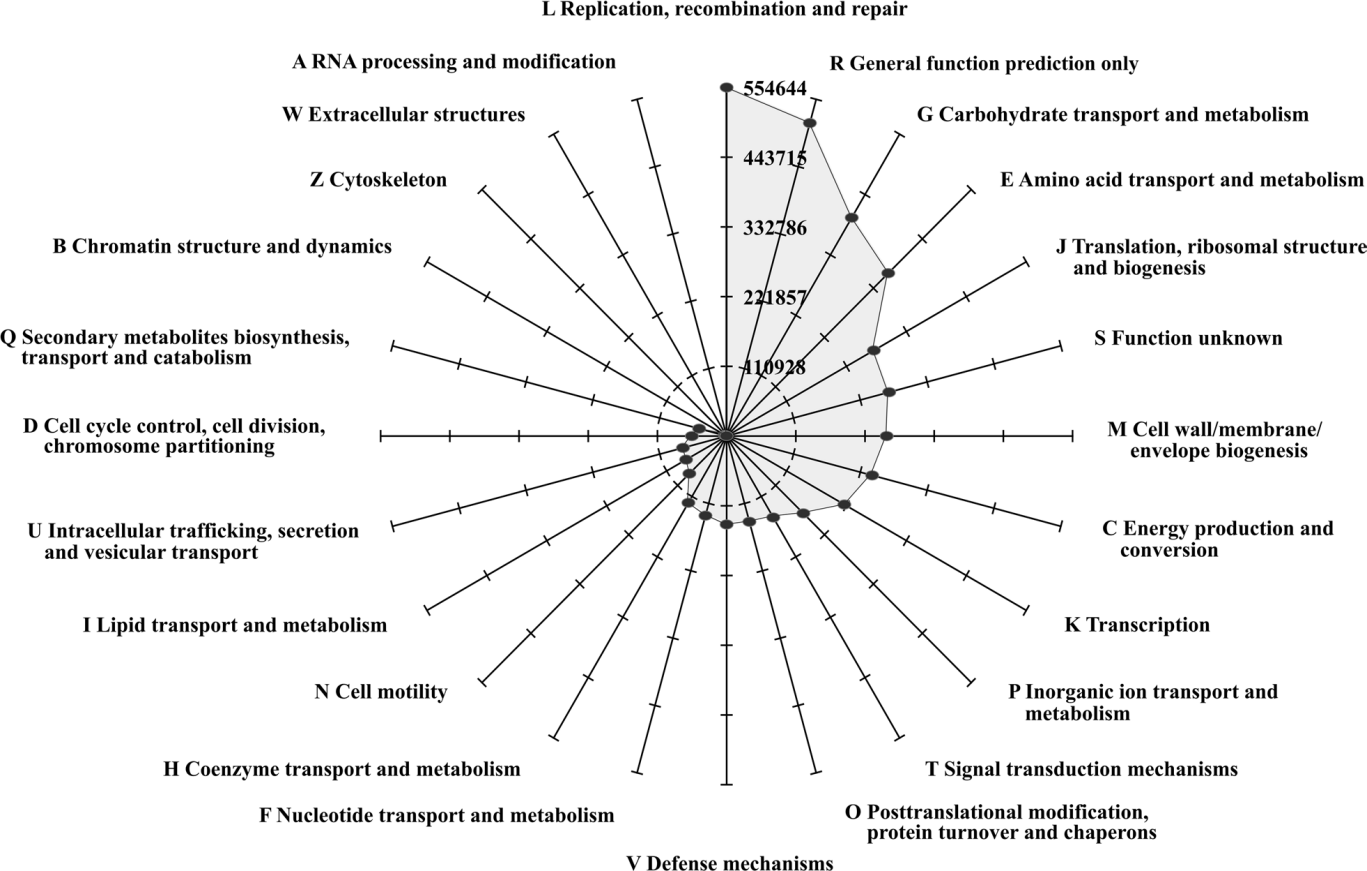
Classification of biogas fermenter metagenome sequences according to Clusters of Orthologous Groups of proteins (COG). Each *line* represents the respective category as labeled at the outside. *Numbers* indicate the counts of metagenome sequences assigned to the corresponding COG category

Overall, 3,777,965 metagenome sequences (58.1 % of all sequences obtained) were assigned to Clusters of Orthologous Groups of proteins (COG), which are subdivided into functional categories (Fig. 4). Some categories, such as ‘carbohydrate transport and metabolism’ (G), ‘amino acid transport and metabolism’ (E) and ‘energy production and conversion’ (C) are well covered by metagenome sequences. They are of particular interest, since during the conversion of biomass to methane initially complex polymers are broken down to oligo- and monomers. Within the functional category ‘carbohydrate transport and metabolism’, assignments to cellobiose phosphorylase (COG3459), glucosidase (COG0366), and cellulase/cellobiase CelA1 (COG5297) indicate the potential of the thermophilic biogas community to degrade cellulose (Table 1).

**Table 1:** Clusters of Orthologous Groups of proteins (COG) associated with the degradation of carbohydrates and proteins and with methanogenesis identified in the metagenome dataset of the thermophilic biogas microbiome

Environmental gene tag		Assigned sequences	Environmental gene tag		Assigned sequences
COG accession	Function		COG accession	Function	
*Polysaccharide degradation and metabolism*			*Methanogenesis—hydrogenotrophic pathway*		
COG0366	Glucosidase	12,524	COG1229	Formylmethanofuran dehydrogenase, subunit A	839
COG1472	Periplasmic beta-galactosidase and related glycosidases^a^	11,683	COG2218	Formylmethanofuran dehydrogenase, subunit C	541
COG3459	Cellobiose phosphorylase	10,420	COG2191	Formylmethanofuran dehydrogenase, subunit E	371
COG1501	Alpha-glycosidase, GH31 family	5616	COG1153	Formylmethanofuran dehydrogenase, subunit D	212
COG2730	Aryl-phospho-beta-D-glycosidase BglC, GH1 family	2588	COG2037	Formylmethanofuran: H4MPT formyltransferase	269
COG1874	Beta-galactosidase GanA	1714	COG3252	Methenyl-H4MPT cyclohydrolase	227
COG2160	L-arabinose isomerase	1539	COG1962	H4MPT S-methyltransferase, subunit H	246
COG3345	Alpha-galactosidase	1385	COG4064	H4MPT S-methyltransferase, subunit G	181
COG1904	Glucuronate isomerase	1030	COG4059	H4MPT S-methyltransferase, subunit E	167
COG3325	Chitinase, GH18 family	958	COG4061	H4MPT S-methyltransferase, subunit C	151
COG3405	Endo-1,4-beta-D-glucanase Y^b^	854	COG4060	H4MPT S-methyltransferase, subunit D	150
COG4124	Beta-mannanase^c^	152	COG4063	H4MPT S-methyltransferase, subunit A	121
COG5297	Cellulase/cellobiase CelA1	136	COG4062	H4MPT S-methyltransferase, subunit B	14
COG0726	Peptidoglucan/xylan/chitin deacetylase, PgdA/CDA1 family	5831	COG4218	H4MPT S-methyltransferase, subunit F	8
COG3507	Beta-xylosidase	4780			
GOG3693	Endo-1,4-beta-xylanase, GH 35 family	3797	*Methanogenesis—acetoclastic pathway*		
GOG4213	ABC-type xylose transport system, periplasmic component	1628	COG1614	CO dehydrogenase/acetyl-CoA synthase, subunit β	510
GOG2115	Xylose isomerase	1591	COG1456	CO dehydrogenase/acetyl-CoA synthase, subunit γ (corrinoid Fe-S protein)	353
COG5434	Polygalacturonase	1023	COG2069	CO dehydrogenase/acetyl-CoA synthase, subunit δ (corrinoid Fe-S protein)	214
COG3867	Arabinogalactan endo-1,4-beta-galactosidase	823	COG1152	CO dehydrogenase/acetyl-CoA synthase, subunit α	198
COG3866	Pectate lyase	541	COG1880	CO dehydrogenase/acetyl-CoA synthase,subunit ε	55
COG4677	Pectin methylesterase and related acyl-CoA thioesterases	228			
COG2132	Multicopper oxidase with three cupredoxin domains (includes cell division protein FtsP and spore coat protein CotA)	423	*Methanogenesis—hydrogenotrophic and acetoclastic pathway*		
			COG4058	Methyl coenzyme-M reductase, subunit α	719
			COG4054	Methyl coenzyme-M reductase, subunit β	550
*Protein degradation*			COG4057	Methyl coenzyme-M reductase, subunit γ	270
COG0740	ATP-dependent protease ClpP, protease subunit	5178	COG4055	Methyl coenzyme-M reductase, subunit D	194
			COG4056	Methyl coenzyme-M reductase, subunit C	107

^a^GH3 family

^b^GH8 family

^c^GH26 family

Peptidoglucan/xylan/chitin deacetylase (COG0726), endo-1,4-beta-xylanase GH35 (COG3693), beta-xylosidase (COG3507), ABC-type xylose transport system (COG4213), and pectate lyase (COG3866) represent enzymes/proteins involved in the degradation of the plant cell wall components hemicellulose, xylan and pectin. Assignments to the formyl-methanofuran dehydrogenase (FMD) subunits ABCD (COG1229, COG1029, COG2218, COG1153), catalyzing the reversible reduction of CO_2_ to N-formyl-methanofuran, were observed, with the latter being an intermediate of hydrogenotrophic methanogenesis.

Characterization of assignments corresponding to the functional categories G, E, and C on the taxonomic level revealed that *Defluviitoga*, *Clostridium*, *Halothermothrix*, and *Tepidanaerobacter* were the dominant genera within these functional categories. However, it should be noted that the majority of determined gene sequences was not assignable to a certain microbial genus or species, as also found for the microbial 16S rRNA sequences and corresponding genes.

To provide insights into the potential of hydrolytic bacteria to degrade plant material, namely the carbohydrate polymers within the biomass, metagenome sequences were compared to the carbohydrate-active enzyme database (CAZy) applying the annotation web-server dbCAN [30] implemented in MGX. A total of 126 glycosyl hydrolase (GH) and 65 carbohydrate binding module families (CBM) were assigned. GH catalyze the hydrolysis of glycosidic bonds in complex carbohydrates, whereas CBMs are the non-catalytic modules of the carbohydrate-active enzymes that are required for substrate-binding. Table 2 summarizes the 15 most abundant GH and CBM modules encoded in the metagenome of the thermophilic biogas community. Inspection of the putative CAZymes revealed that GH families 43, 10 and 5 representing endo-xylanases acting on hemicellulose are highly abundant. Moreover, GH families 2 and 3, described to comprise mannosidases and galactosidases involved in the degradation of mannan within hemicellulose, were also represented. GH families 9 and 48 acting on cellulose were found less frequently compared to the above-mentioned families. However, the analyzed metagenome encodes a variety of enzymes that digest carbohydrate polymers and other oligosaccharides. Among the identified CBM modules, the CBM50-type, predicted to bind chitin, was surprisingly highly abundant in the metagenome dataset followed by CBM 44, 9, 6 and 22, which were described to bind hemicellulose and cellulose.

**Table 2:** The 15 most abundant glycoside hydrolase (GH, left table) and carbohydrate binding modules (CBM, right table) families of the thermophilic biogas-producing microbial community as analyzed by means of the carbohydrate-active enzyme database (CAZy) annotation web-server dbCAN

Predicted CAZy glycoside hydrolase (GH) family	Number of sequences in the combined microbial metagenome	Predicted CAZy carbohydrate binding modules (CBM) family	Putative binding substrate	Number of sequences in the combined microbial metagenome
GH13	12,265	CBM50	Chitin	10,403
GH94	8720	CBM44	Xylan	4032
GH43	5793	CBM48	α-glucan	2030
GH109	5696	CBM32	β-mannans	1943
GH2	5506	CBM35	β-glucans, pectins, mannans, gluco- and galacturonans	1921
GH3	5075	CBM9	Crystalline cellulose	1906
GH23	4155	CBM6	Cello-oligosaccharides, laminarin	1842
GH31	3912	CBM22	Amorphous celluloses and insoluble and soluble xylan	1823
GH18	3896	CBM34	Starch	1405
GH10	3385	CBM16	Cellulose and glucomannan	1299
GH4	3015	CBM41	α-glucans, amylose, amylopectin, pullulan	1114
GH51	2983	CBM66	Fructans	1000
GH57	2499	CBM40	Sialic acid	715
GH95	2408	CBM4	Amorphous cellulose	687
GH5	2257	CBM67	Rhamnose	678

The key enzymes of the methanogenesis pathway were assigned to the functional COG category ‘coenzyme transport and metabolism’ (H), including archaeal subunits of methyl coenzyme M reductase. Most of the hits assigned to the category H originate from the genus *Methanoculleus*.

### Diversity of facultative anaerobic bacteria as determined by standard plating approaches and subsequent MALDI-TOF MS-based identification

For a first cultivation-based access to the microbial diversity, a well-established commercial pipeline for the microbiological routine analytics of environmental samples was applied consisting of plating dilution series on traditional standard media and culturing at anoxic or microoxic conditions combined with MALDI-TOF MS identification [isolation strategies (1)–(2)]. This approach allowed the identification of aerobic, microaerophilic and (facultative) anaerobic bacteria with the help of reference strains from culture collections and the determination of corresponding cell abundance within one sample (Additional file 1: Figure S4).

Overall, 20 bacterial genera of seven classes were detected in samples of the fermentation substrate, the main fermenter, and the digestate with cell numbers between 10^2^ and 10^8^ colony forming units (CFU) per gram sample, which is in the expected range of 10^4^–10^8^ CFU per gram sample [61, 62] (Table 3; Additional file 1: Figure S4). In the main fermenter sample, members of the class *Bacilli* and, within this, the genera *Bacillus* and *Ureibacillus* were most prevalent with abundances of up to 5 × 10^7^ CFU g^−1^; with abundances between 10^3^ and 10^5^ CFU g^−1^ members of the class *Clostridia* were detected less frequently in this assay.

**Table 3:** Summary of pure isolates obtained from the thermophilic biogas plant applying different isolation strategies (1)–(11) assigned to characterized reference species by means of their 16S rRNA gene sequence similarity using the EzTaxon identification tool; metagenomic and metatranscriptomic sequences were mapped on a reference strain 16S rRNA gene sequence applying the gsMapper program

Closest related NCBI GenBank entry				Isolates from the thermophilic biogas plant								Number of metagenome sequences mapped on the 16S rRNA^b^	Number of metatranscriptome sequences mapped on the 16S rRNA^c^
Class	Family	Species	Accession number	16S rRNA identity (%)	Number of isolates and/or colony forming units (CFU)	Isolate designation	NCBI Genbank entry	Cellulose degrader	Main substrate	Fermentation products	Isolation strategy^a^		
Cellulolytic *Bacteria*													
*Clostridia*	*Lachnospiraceae*	*Herbinix hemicellulosilytica*	LN626355	100.0	1 isolate	T3/55^T^	LN626355	Yes	Cellobiose	Et, Ac, Pr	4	17	1552
				96.4	8 isolates	SD1D	LN626359	Yes	Cellobiose	Et, Ac, Pr	4, 5	17	858
	*Ruminococcaceae*	*Clostridium cellulosi*	L09177	98.9	14 isolates	DG5	LN881577	Yes	Cellobiose	Et, Ac	3, 4	0	1831
		*Clostridium clariflavum*	CP003065	100.0	34 isolates	K50/5	LN881585	Yes	Cellulose	NA^d^	3, 4	862	9152
		*Clostridium stercorarium*	CP004044	99.3	13 isolates	Neu14	LN881576	Yes	Cellobiose	NA	3	28	12,099
		*Clostridium thermocellum*	CP000568	99.9	27 isolates	HAW2/1	HG917924	Yes	Cellulose	Et, Ac, Ip	3, 4, 5	1279	7814
Acidogenic/acetogenic *Bacteria*													
*Bacilli*	*Bacillaceae*	*Bacillus cereus*	–	NA	2 × 10^7^ CFU g^−1^			(No)	NA	NA	1	NA	NA
		*Bacillus licheniformis*	AE017333	99.9	5 × 10^7^ CFU g^−1^, 9 isolates	L2C	KT351634	(No)	NA	NA	1, 8a, 8b, 9	2	2696
		*Bacillus oleronius*	–	NA	1 × 10^7^ CFU g^−1^			(No)	NA	NA	2	NA	NA
		*Bacillus thermoamylovorans*	L27478	99.5	1 × 10^7^ CFU g^−1^, 7 isolates	Neu19	LN881587	No	Starch	Et, Ac, Fo	2, 3, 4, 8c, 8d	1	5070
		*Bacillus coagulans*	AB271752	99.6	2 isolates	M1A	KT351636	NA	NA	NA	8a	3	2566
		*Bacillus infernus*	U20385	99.9	3 isolates	E2C	KT351638	NA	NA	NA	8d	3	3989
		*Geobacillus thermodenitrificans*	CP000557	100.0	2 isolates	J2B	KT351633	NA	NA	NA	8a, 8b	0	4181
	*Enterococcaceae*	*Enterococcus faecium*	–	NA	<10^2^ CFU g^−1^			(No)	NA	NA	2	NA	NA
	*Paenibacillaceae*	*Aneurinibacillus* spp.	–	NA	<10^3^ CFU g^−1^			(No)	NA	NA	2	NA	NA
		*Paenibacillus barengoltzii*	AY167814	99.1	6 isolates	YP4-6A	KT351639	NA	NA	NA	8a	0	1788
	*Planococcaceae*	*Ureibacillus thermosphaericus*	AB101594	100.0	3 × 10^7^ CFU g^−1^, 3 isolates	A6A	KT351635	NA	NA	NA	1, 9	2	4376
*Clostridia*	*Clostridiaceae*	*Clostridium isatidis*	X98395	99.9	13 isolates	MV1	LN881568	No	Glucose	NA	3, 4	738	2404
				99.3	2 isolates	RX1	LN881572	No	Glucose	NA	3	65	2027
		*Clostridium perfringens*	–	NA	<10^5^ CFU g^−1^			(No)	NA	NA	1	NA	NA
		*Clostridium putrefaciens*	Y18177	94.2	7 isolates	Neu23	LN881581	No	Cellobiose	NA	4, 8a	286	4415
				94.2	17 isolates	N1F	KT351631	NA	NA	NA	8a, 8b	281	4347
		*Clostridium sporogenes*	–	NA	<10^5^ CFU g^−1^			(No)	NA	NA	1	NA	NA
		*Clostridium thermopalmarium*	X72869	99.5	1 isolate	Neu4	LN881579	No	Glucose	NA	4	11	1180
		*Lutispora thermophila*	AB186360	100.0	1 isolate	XV1	LN881567	No	Glucose	NA	3	3	2000
		*Tepidimicrobium ferriphilum*	AY656718	97.4	1 isolate	GRX2	LN881569	No	Glucose	NA	3	9	6191
				97.4	1 isolate	GRM1	LN881570	No	Glucose	NA	3	10	6204
				97.5	1 isolate	Z2-16	KT351637	NA	NA	NA	8a	9	6123
				98.7	1 isolate	GRC5	LN881575	No	Glucose	NA	3	20	5958
				96.2	1 isolate	D1	KT274718	NA	Glucose^e^	Ac	6	5	6609
				94.4	1 isolate	GRC3	LN890940	No	Glucose	NA	3	12	7329
		*Tepidimicrobium xylanilyticum*	EF522948	99.7	1 isolate	GRC4	LN881574	No	Glucose	NA	3	27	4322
				96.7	2 isolates	GRC1	LN881571	No	Glucose	NA	3	13	8131
				96.3	1 isolate	XV2	LN881586	No	Glucose	NA	3	22	5549
	*Defluviitaleaceae*	*Defluviitalea saccharophila*	HQ020487	96.5	1 isolate	GRX3	LN881573	No	Glucose	NA	3	12	2923
	*Peptococcaceae*	*Desulfotomaculum australicum*	M96665	99.5	1 isolate	L14	KT274713	No	Lactic acid	Ac	6	1	3784
	*Ruminococcaceae*	*Clostridium straminisolvens*	BAVR01000144	98.3	3 isolates	Neu18	LN881580	No	Cellobiose	NA	4	37	9436
		*Clostridium caenicola*	AB221372	97.6	3 isolates	Neu21	LN881582	No	Glucose	NA	3, 4	9	8238
		*Clostridium thermosuccinogenes*	Y18180	99.8	14 isolates	Iso4/1b	LN881583	No	Glucose	NA	3, 4	7	6742
	*Thermoanaerobacteraceae*	*Tepidanaerobacter syntrophicus*	AB106353	96.6	1 isolate	AS34	KT274714	NA	Amino acids^f^	Ac, Pr	6	23	8696
				96.2	1 isolate	AS46	KT274715	NA	Amino acids^f^	Ac, Pr	6	43	12,295
	*Incertae sedis*	*Sporanaerobacter acetigenes*	AF358114	98.7	1 isolate	XP2-13-3	KT351640	NA	NA	NA	8b	0	1715
		*Thermoanaerobacterium thermosaccharolyticum*	CP002171	99.2	24 isolates	Iso6/1b	LN881584	No	Glucose	NA	3	0	1838
				99.9	1 isolate	Gluc2	KT274716	NA	Glucose	Ac, Bu, La	7	0	3000
				99.9	1 isolate	Gluc4	KT274717	No	Glucose	Ac, Bu, La	7	0	2881
		*Tissierella creatinini*	FR749955	96.2	1 isolate	DG3	LN881578	No	Glucose	NA	3	1	2229
	Unclassified	*Proteiniborus ethanoligenes*	EF116488	95.9	2 isolates	BA2-13	KT351641	NA	NA	NA	8e	0	1809
*Thermotogae*	*Petrotogaceae*	*Defluviitoga tunisiensis*	FR850164	99.9	7 isolates	L3	KT274706	No	Lactic acid	Ac	6	968	115,754
				99.3	1 isolate	AS30	KT274709	NA	Amino acids^f^	Ac	6	201	39,551
Methanogenic *Archaea*													
*Methanobacteria*	*Methanobacteriaceae*	*Methanothermobacter marburgensis*	CP001710	99.7	1 isolate	Viersen-HAW	KU667127	(No)	H_2_/CO_2_	NA	10	13	32
		*Methanothermobacter wolfeii*	AB104858	100.0	1 isolate	SIV6	KT368944	(No)	H_2_/CO_2_	NA	11	39	60
*Methanomicrobia*	*Methano*-*microbiaceae*	*Methanoculleus thermophilus*	AB065297	100.0	3 isolates	V2.1	KT368945	(No)	H_2_/CO_2_	NA	11	389	35,733

*Et* ethanol; *Ac* acetic acid; *Pr* propionic acid; *Ip* isopropanol; *Fo* formic acid; *Bu* butyric acid; *La* lactic acid

^a^For details, refer “Methods” section

^b^In total, 18,817 16S rRNA gene sequences

^c^In total, 532,381 16S rRNA sequences

^d^Not analyzed

^e^Formation of acetic acid in medium DSMZ 1328 [104] with 0.5 % (w/v) glucose

^f^Minimal medium (modified DSMZ 287 [36]) containing alanine, threonine, serine, glutamic acid, cysteine and methionine

As expected, most of the species detected in the main fermenter were not detectable in the primary fermentation substrate, i.e. swine manure. Overall, with species from six classes, the isolates derived from the presumably mesophilic manure sample were more diverse than the isolates from the thermophilic fermenter fluid indicating the selection and enrichment of particular species within the thermophilic main fermenter of the biogas plant. However, also within the manure, members of the class *Bacilli* were most prevalent but belong to genera and species that differed from the fermenter fluid. As an example, *Lactobacillus* spp. were found with abundances of up to 4 × 10^7^ CFU g^−1^. In addition, single species from the classes *Actinobacteria*, *Bacteroidia*, *Flavobacteria*, *γ*-*Proteobacteria*, and, with lower abundances, from the class *Clostridia* were cultivated from the manure sample.

The microbial community in the unheated digestate tank at the end of the anaerobic digestion and biomethanation process differed from the one of the process fluid from the main fermenter. In addition to nine species also present in the fermentation substrate and/or the main fermenter fluid, eight species were detected exclusively in the digestate. The most prevalent class was again the class *Bacilli*, followed by *Clostridia* and *Bacteroidia*. Also members of the classes *β*- and *γ*-*Proteobacteria* were found in the digestate.

The neurotoxic *Clostridium botulinum* was never detected, either by cultivation or by testing for the toxins itself using the established method published previously [63] (data not shown). Moreover, no extended-spectrum β-lactamase producing (ESBL) strains were cultivated (data not shown). The pathogenic *Clostridium perfringens* was detected throughout the biogas plant with abundances between 10^4^ and 10^5^ CFU g^−1^. In addition, pathogenic *Salmonella* species were detected in all samples after enrichment (data not shown). Out of the digestate sample, *S. enterica* ssp. *enterica* was cultivated and identified as serovar *Thyphimurium*.

As expected, methanogenic *Archaea* were not cultivated by any of the plating cultivation approaches applied in the context of MALDI-TOF MS identification approach. In contrast, several fungi were cultivated from all samples. Besides some unknown fungal species, *Pichia fermentans* (ascomycetes) was found in the fermentation substrate, while *Rhodotorula mucilaginosa* (basidiomycetes) was identified in the main fermenter and *Scopulariopsis brevicaulis* (ascomycetes) in the digestate. In addition, a number of bacteria specified as “unknown” in the MALDI-TOF MS analysis was detected throughout all samples.

### Cultivation-based estimation of the diversity of cellulolytic/hydrolytic microorganisms

In order to isolate characteristic species for distinct trophic groups, namely (hemi-) cellulolytic, acidogenic/acetogenic, and methanogenic microbial species, more sophisticated isolation strategies were applied. An overview on all isolates and their taxonomic affiliation is given in Table 3 as well as the abundance of their 16S rRNA genes in the microbial metagenome and metatranscriptome datasets.

With cellulose or hemicellulose as main carbon source in the different growth media, 172 pure thermophilic isolates were obtained from the thermophilic model biogas plant applying different strictly anoxic isolation strategies, namely strategies (3)–(5). Among them, 97 isolates (56 %) were able to degrade crystalline cellulose, i.e. truly cellulolytic. With enrichment substrates such as xylan or other hemicelluloses, some hydrolytic bacteria were isolated that were not able to degrade crystalline cellulose. Especially mixed substrates (hemicelluloses) with a variety of different polysaccharides promote a high diversity of the isolates. All isolates belong to the phylum *Firmicutes* and, within this phylum, to the classes *Clostridia* and *Bacilli*.

Among the *Clostridia*, several isolates were obtained featuring high 16S rRNA sequence similarity (≥98.9 %) with the well characterized cellulolytic bacteria *Clostridium cellulosi*, *C. stercorarium*, *C. clariflavum*, and *C. thermocellum*. From these species, *C. thermocellum* and *C. clariflavum* 16S rRNA gene sequences were found to be most prevalent in the metagenomic dataset as indicated by 1279 and 862 mapped sequences (Table 3). In contrast, in the metatranscriptome dataset, the *C. stercorarium* 16S rRNA gene sequences were most abundant (12,099 sequences) followed by *C. clariflavum* (9152 sequences) and *C. thermocellum* (7814 sequences). It needs to be mentioned that the majority of the recruited metagenome and metatranscriptome sequences were mapped to a conserved segment of the reference species’ 16S rRNA gene sequence eventually leading to an overestimation of the abundance of particular species. Referring to this, the resolution of metagenome analyses is limited by the relatively short Illumina read lengths which does not cover the complete 16S rRNA gene. However, these results supported the assumption that *Clostridium* species predominate in the anaerobic digestion of cellulosic and highly complex organic compounds.

In addition to representatives of the genus *Clostridium*, some cellulolytic bacteria were isolated, forming a subcluster within the family *Lachnospiraceae*. From these isolates strain T3/55^T^ was recently described as a new species of a new genus, *Herbinix hemicellulosilytica* [35]. The major end-products of cellulose metabolism were acetic acid, ethanol and propionic acid. Furthermore, *H. hemicellulosilytica* str. T3/55^T^ exhibited a high hemicellulolytic activity on various xylans. The genome sequence of this isolate was used for recombinant expression and characterization of biomass degrading enzymes [49].

### Cultivation-based estimation of the diversity of acidogenic/acetogenic microorganisms

Using a minimal medium supplemented with primary fermentation products as carbon source, namely glucose, amino acids, and lactic acid [isolation strategies (6) and (7)], in total 14 acid forming isolates were identified belonging to the classes *Clostridia* and *Thermotogae* and the corresponding families *Clostridiaceae*, *Peptococcaceae*, *Petrotogaceae*, *Thermoanaerobacteraceae* and *Thermoanaerobacterales* Family III *incertae sedis* (Table 3). Within the class *Clostridia*, two strains (str. Gluc2 and str. Gluc4) isolated by plating on anoxic medium were forming acetic acid, butyric acid, and lactic acid from glucose. Both were identified as *Thermoanaerobacterium thermosaccharolyticum*. The other isolates were obtained by using the deep agar shake method. Strains AS34 and AS46 showed a similarity of >96 % based on their 16S rRNA gene sequences to the genus *Tepidanaerobacter* and were able to produce acetic acid and propionic acid from a mixture of six amino acids. Furthermore, two acetic acid forming strains, str. D1 and str. L14, affiliated to *Tepidimicrobium ferriphilum* and *Desulfotomaculum australicum*, respectively, were isolated. Most isolates, i.e. eight strains, were related to *Defluviitoga tunisiensis* and were obtained from enrichment cultures grown on Na^+^-DL-lactate, succinate, or a mixture of amino acids. All isolated strains formed acetic acid from the carbon source provided.

In the metagenome and metatranscriptome datasets, *D. tunisiensis* was represented by 968 and 115,754 16S rRNA gene sequences (Table 3) indicating a predominance and metabolic activity in the thermophilic biogas plant. However, in this context the applied approach for analysis of the metagenome and metatranscriptome datasets is suited to provide first hints but not to prove certain ecological relationships.

Applying further cultivation strategies using various media targeting different anaerobic and facultative anaerobic bacteria [isolation strategies (8)–(9)], 56 isolates were obtained. These isolates represented twelve different strains of genera which are known to contain acidogenic/acetogenic bacteria. All isolates belong to the classes *Clostridia* and *Bacilli*, and within these classes to the families *Bacillaceae*, *Paenibacillaceae*, *Planococcaceae*, *Clostridiaceae*, *Tissierellales* Family XI *incertae sedis* and to one recently unclassified family (Table 3).

### *Cultivation*-*based estimation of the diversity of methanogenic* Archaea

In contrast to the fermentative *Bacteria*, methanogenic *Archaea* from three different species were isolated in only five specimens (Table 3). As determined by 16S rRNA gene sequencing, the isolates were assigned to *Methanothermobacter marburgensis*, *Mt. wolfeii*, both members of the class *Methanobacteria*, and *Methanoculleus thermophilus* (class *Methanomicrobia*). All species were characterized as hydrogenotrophic thermophilic methanogenic *Euryarchaeota*, whereby *Methanothermobacter* spp. are rod-shaped methanogens with a length of up to approximately 6 µm (Additional file 1: Figure S5). In contrast, the cells of *Mc. thermophilus* are irregular cocci of up to 1.3 µm in diameter that are non-motile.

Mapping of the corresponding 16S rRNA gene sequences in the metagenome and metatranscriptome datasets underlined a certain prevalence of *Mc. themophilus* as indicated by 389 and 35,733 mapped sequences, respectively.

### Diversity of the bacterial and archaeal isolates and community profiles

Within this study, 52 different isolates (according to their 16S rRNA gene sequences) were obtained, which show closest affiliation to 32 validly named species, i.e. 29 species of the domain *Bacteria* and three species of the domain *Archaea* (Table 3; Additional file 1: Figure S6). The bacterial isolates belonged to the classes *Clostridia*, *Bacilli*, and *Thermotogae*, while the archaeal isolates comprised the orders *Methanobacteria* and *Methanomicrobia.*

A large and very diverse group of isolates belonged to the class *Clostridia*. Altogether, the obtained *Clostridia* isolates showed closest affiliation to 23 different characterized species. Among them, most of the isolates represent members of the family *Ruminococcaceae* closely related to *C. cellulosi*, *C. thermocellum*, and *C. clariflavum* (see also Table 3). All three species were described as efficient lignocellulosic biomass degraders [64, 65]. Taxonomic classification based on metagenomic 16S rRNA genes of the analyzed biogas microbial community also showed that the class *Clostridia*, namely species from the genera *Clostridium* sensu stricto and *Clostridium* cluster III, were highly abundant in the analyzed biogas plant.

Furthermore, a total of eleven different bacterial strains belonging to the class *Bacilli* were isolated. However, the taxonomic profile based on metagenomic and metatranscriptomic 16S rRNA gene fragments showed that the *Bacilli* group is not very abundant in the thermophilic fermenter and only represents 0.1 and 0.2 % of 16S rRNA genes and transcripts derived from the metagenome and metatranscriptome datasets. Hence, it can be assumed that these microorganisms play a minor role in thermophilic anaerobic biomass degradation.

Two phylogenetically different groups of isolates were isolated classified as *Defluviitoga tunisiensis* representing an isolated branch of the phylogenetic tree due to their taxonomic affiliation to the phylum *Thermotoga* (Table 3). Approximately 7.1 % of the metagenomic 16S rRNA gene sequences were assigned to this phylum demonstrating its importance within the studied thermophilic microbial community responsible for the biomethanation process (Fig. 3).

Within the domain *Archaea*, isolates closely related to members of the orders *Methanobacteria* and *Methanomicrobia* were obtained. Within these groups two *Methanothermobacter* species and one *Methanoculleus* species were isolated, respectively. These archaeal representatives belong to the most abundant methanogens prevailing in the analyzed thermophilic microbial community as deduced from the taxonomic profile based on metagenome sequence data.

### Prevalence of three hydrolytic isolates in the thermophilic biogas microbiome as determined by fragment recruitment analysis

To determine the degree of relatedness of the exemplarily chosen hydrolytic isolates representing two functional groups of the biogas process, namely cellulolytic and acidogenic/acetogenic *Bacteria* (Table 3), fragment recruitment was performed (Table 4; Additional file 1: Figure S7). Therefore, three biogas process-relevant microbial community members isolated from the analyzed digester, namely *Clostridium* resp. *Ruminiclostridium cellulosi* str. DG5 (*Ruminococcaceae*, genus denomination and affiliation currently under revision) *Herbinix hemicellulosilytica* str. T3/55^T^ (*Lachnospiraceae*) and *Defluviitoga tunisiensis* str. L3 (*Petrotogaceae*), were selected.

**Table 4:** Number of metagenome sequences representing the thermophilic biogas microbial community mapped on the reference genome sequences of three isolates

Number of metagenome sequences mapped on the reference genome [%]			
Sequence identity (%)	*Clostridium cellulosi* str. DG5	*Herbinix hemicellulosilytica* str. T3/55^T^	*Defluviitoga tunisiensis* str. L3
100	0.005	0.031	3.966
99	0.013	0.074	6.402
98	0.023	0.106	6.952
97	0.031	0.144	7.238
96	0.036	0.173	7.443

The genome of the *C. cellulosi* str. DG5 was recently established and published [48]. This isolate was predicted to grow on various poly- and monosaccharides, since all necessary genes were identified in the DG5 genome. Moreover, the strain DG5 encodes all known genes required for growth on cellulose, but does not contain genes encoding cellulosome components. *H. hemicellulosilytica* str. T3/55^T^ was recently described as bacterial strain involved in thermophilic degradation of lignocellulosic biomass such as crystalline cellulose [35]. Analysis of the *H. hemicellulosilytica* str. T3/55^T^ genome showed that it harbors genes encoding a cellulolytic system consisting of three cellulases, one endoglucanase (glycoside hydrolase family 9, GH9) and two cellobiohydrolases (GH5 and GH48) presumably degrading cellulose [49]. Ethanol, acetic acid and propionic acid are the major fermentation end-products.

The genome of the strain *D. tunisiensis* str. L3 harbors genes predicted to facilitate utilization of a variety of complex polysaccharides including cellulose, chitin, and xylan indicating the strain’s contribution to the hydrolysis step of the anaerobic digestion chain [27]. Ethanol, acetate, H_2_, and CO_2_ were predicted to be fermentation end-products [52]. The latter three metabolites represent important substrates for methanogenic *Archaea*. Thus, it can be hypothesized that, at least in the investigated thermophilic biogas plant, *D. tunisiensis* probably forms a close physiological or syntrophic relationship with the identified hydrogenotrophic methanogens of the genera *Methanothermobacter* and *Methanoculleus* as it was shown in a comparable way, for example, for *Thermotoga maritima* and *Methanocaldococcus jannaschii* [66].

As indicated by its 16S rRNA gene and transcript abundance, *D. tunisiensis* str. L3 is present in the thermophilic biogas plant in comparatively high amounts and metabolically active. However, *C. cellulosi* str. DG5 and *H. hemicellulosilytica* str. T3/55^T^ were detected only rarely in the metagenome and metatranscriptome datasets. To verify the mapping results for the 16S rRNA gene, the metagenome sequences obtained for the thermophilic biogas plant were mapped onto the genome sequences of *C. cellulosi* str. DG5, *H. hemicellulosilytica* str. T3/55^T^ and *D. tunisiensis* str. L3 (Table 4; Additional file 1: Figure S7). It could be confirmed, that *C. cellulosi* str. DG5 and *H. hemicellulosilytica* str. T3/55^T^ genes were present but underrepresented in the biogas plant microbiome. In case of *C. cellulosi* str. DG5, 2043 metagenome sequences (0.03 % of all metagenome sequences) were recruited with at least 97 % sequence identity (Table 4; Additional file 1: Figure S7a). Mapping results for the strain T3/55^T^ showed 9329 recruited metagenome sequences (0.1 % of all metagenome sequences) (Table 4; Additional file 1: Figure S7b). In contrast, in case of the *D. tunisiensis* str. L3 it could be shown that this species is highly abundant within the biogas microbiome. Approximately 7.2 %, i.e. 470,497 sequences, of all metagenome sequences were mapped onto the genome sequence of str. L3, with 4.0 % of them featuring 100 % identity to the reference (Table 4; Additional file 1: Figure S7c). These findings confirmed that the strain *D. tunisiensis* plays an important role within the thermophilic anaerobic digestion and the biogas production process in the biogas plant studied.

## Discussion

### The investigated thermophilic biogas plant—suited to serve as representative biogas plant?

Anaerobic digestion and biomethanation of organic material under thermophilic conditions is assumed to be advantageous compared to a mesophilic temperature regime. In the literature, several examples are documented where thermophilic biomethanation resulted in higher organic degradation and methane yield than mesophilic biomethanation, as shown in case of anaerobic digestion of cattle manure in laboratory scaled experiments [18]. Surprisingly, in Germany, thermophilic industrial-scaled biogas plants are rare compared to mesophilic ones [16], since operation and control of thermophilic processes are demanding [67].

Generally, simple one stage agricultural biogas plants with a round concrete reservoir and an internal mixer are commonly operated at mesophilic temperatures between 37 and 41 °C and with an average organic loading rate (OLR) of 3 kg _VS_ m^−3^ d^−1^ and a HRT of 80 d [68]. In contrast, the thermophilic biogas plant investigated in this study executed an OLR of 8 kg _VS_ m^−3^ d^−1^ and a HRT of 19.8 d and, thus, had a several hundred percentage higher time–space yield. On average, the analyzed biogas plant permanently produced 175 KW_el_ (approx. 4 250 kW h d^−1^) and 190 kWh of heating energy corresponding to 99 % of the German general planning standard [69]. In addition, the electricity generation resembles to a daily gas production of about 728 m^3^ d^−1^ or a specific gas production rate of about 0.62 m_biogas_^3^ kg _VS_^−1^ d^−1^ with a methane content of 53–54 %. All these data indicate that the analyzed thermophilic biogas plant showed a very good performance during the time period when sampling occurred.

One explanation for this excellent efficiency of gas production is believed to be the thorough mixing of the fermenters by pumps, the width to height ratio of the cylindrical reactors, and the thermophilic temperature. It is well-established that with temperature the partial H_2_ pressure will increase in a closed fermenter. Due to thermodynamic reasons, the H_2_ concentration in a closed thermophilic fermenter is typically increased five to tenfold [70]. That depends on the height and the temperature. According to the equation of free energy, ∆*G*_f_ = ∆H_f_ − T∆S, whereby ∆H_f_ is the free enthalpy or heat of formation, T is the absolute temperature, and ∆S is the entropy value [71], and the temperature dependency of the entropy term (T∆S), at thermophilic conditions the ∆*G*_f_ will be more negative and therefore the whole process is much more exergonic. This is in accordance with the good performance of this type of thermophilic biogas plant.

### *The thermophilic biogas microbiome*—*the first stage, cellulolytic and hydrolytic* Bacteria

The metagenome sequence analysis from the thermophilic reactor sample revealed that the bacterial phylum *Firmicutes* dominated the biogas community. Additionally, all obtained cellulolytic isolates from the investigated biogas plant belong to the phylum *Firmicutes* and the class *Clostridia*. Although several members of other phyla were detected by metagenome analysis, no cellulolytic species from classes or phyla other than *Firmicutes* were obtained.

In anaerobic environments as prevailing in biogas plants, mainly representatives of the phylum *Firmicutes* are responsible for lignocellulosic biomass degradation [35]. This statement is corroborated by the isolation of representatives exclusively from the phylum *Firmicutes*, when the enrichments were performed on cellulose and hemicellulose. In particular, the results of metatranscriptome and metaproteome analyses of other biogas plants suggested that especially the *Clostridia* provide glycoside hydrolases and therefore species of this class are mainly enriched and isolated on cellulose [19].

In this study, cellulolytic *Clostridia* were isolated frequently; and the genera *Clostridium* cluster III and *Clostridium* sensu stricto belong to the most prominent ones within the bacterial community among the classified 16S rRNA gene sequences. Certainly, the isolated members of these genera such as *C. thermocellum* play a central role in biomass degradation. But the frequent isolation of this species from biogas plants is primarily not only due to the high abundance in the initial sample, but also because this species is easily cultivable under laboratory conditions. Their culturability is undoubtly the major reason for their prominence among the isolated species. Consequently, 90 % of the cellulolytic isolates belonged to already known species.

However, the isolation and characterization of new cellulolytic bacteria able to anaerobically degrade fibrous biomass will help to gain insights into their performance and requirements regarding substrate hydrolysis with the objective to optimize the first phase of biomass decomposition. *Herbinix hemicellulosilytica* str. T3/55^T^ and other cellulolytic isolates are promising candidates for the biotechnological production of novel biomass, especially carbohydrate-degrading enzyme systems that may be applied in the production of fuels, chemicals, and other bio-based materials through the conversion of cellulosic plant material.

Taxonomic characterization of the thermophilic biogas community revealed that in comparison to the mesophilic biogas microbiome [8, 13, 72] members of the bacterial families *Petrotogaceae* (phylum *Thermotogae*) as well as *Halaerobiaceae* (phylum *Firmicutes*) are highly abundant. Thermophilic bacteria have received considerable attention as sources of highly active and thermostable cellulolytic and xylanolytic enzymes. *Petrotogaceae* members from the genus *Defluviitoga* such as *Defluviitoga tunisiensis* str. L3 are described to utilize a large variety of complex carbohydrates including cellobiose, xylan and xylose [52]. Ethanol, acetate, H_2_, and CO_2_ were predicted as possible fermentation end-products. A well characterized *Halocella* species, *Halocella cellulolytica* was described to degrade cellulose forming acetate, ethanol, lactate, H_2_, and CO_2_ as end-products [73]. This species represents another potential key player in the hydrolysis step of the thermophilic digestion process. Moreover, *Halocella* spp. are capable to tolerate high salt concentrations [73] frequently occurring within biogas reactor environments.

Analysis of metatranscriptome sequence data obtained for the thermophilic biogas plant revealed that *Defluviitoga* and *Halocella* genes were highly transcribed indicating high metabolic activity of species belonging to these genera. Moreover, a fragment recruitment approach showed that the *D. tunisiensis* str. L3 genome is almost completely covered with metagenome sequences, with 3.9 % of the recruited reads featuring 100 % identity to the genome sequence of strain L3. This result confirms that strains highly related to the reference strain *D. tunisiensis* L3 play a key role within the community of the thermophilic biogas-production plant.

 In contrast to anaerobic digesters that operated at a mesophilic (35–38 °C) or hyperthermophilic (>65 °C) temperature regime [8, 10, 13], only a few members of the phylum *Bacteroidetes* (class *Bacteroidia*) were detected in the analyzed thermophilic biogas plant operated at 54 °C. Earlier studies, e.g. [14], assumed that under mesophilic conditions *Bacteroidetes* are playing an important role in protein degradation. The substrates applied in this study for anaerobic digestion, namely maize silage (56 % FM) and pig manure (32 % FM), mainly consist of carbohydrates. It remains questionable, which microorganisms are responsible for protein degradation under the thermophilic temperature regime. Potential candidates can eventually be found in the family *Porphyromonadaceae* [74]. However, the species of this family, detected in the metagenome dataset of the analyzed thermophilic biogas plant with 0.33 %, remained unknown due to the lack of closely related reference species.

### *The thermophilic biogas microbiome*—*the second stage, primary and secondary fermenting* Bacteria

Hydrolytic active bacteria produce a broad range of short-chained VFA, mainly with acetate as end-product. Other VFA such as propionate, butyrate, and others are produced in minor amounts depending on the current process conditions and the microbial community characteristics. Acetate is the main precursor for the acetoclastic methanogenesis by methanogenic *Archaea* of the genera *Methanosaeta* and *Methanosarcina*.

However, in the analyzed thermophilic biogas plant, such acetoclastic methanogens were not identified, neither by microbiological nor by molecular approaches. These findings led to the conclusion that the hydrogenotrophic methanogenesis represents the major route for methane synthesis. Consequently, acetate most probably is oxidized to CO_2_, and H_2_ by syntrophic acetate oxidizing bacteria (SAOB). For thermodynamic reasons, acetate oxidation requires a low H_2_ partial pressure. In case of biomethanation, a close cooperation, i.e. syntrophy, of the SAOB with hydrogenotrophic methanogens is required [75].

Recently, quite a few examples for SAOB living in association with hydrogenotrophic methanogens were described, namely *Acetobacterium woodii* and *Methanobacterium* spp., *Clostridium ultunense* and *Methanoculleus* sp., *Thermoacetogenium phaeum* and *Methanothermobacter thermautotrophicus*, *Thermotoga lettingae* and *Methanothermobacter*, *Thermotoga maritima* and *Methanocaldoccocus janaschii*, *Tepidanaerobacter syntrophicus* and *Methanothermobacter thermautotrophicus*, *Syntrophaceticus schinkii* and *Methanoculleus* sp. and *Tepidanaerobacter acetatoxydans* and *Methanoculleus* sp. [76, 77]. The majority of these syntrophic relationships originate from biomethanation systems operating at thermophilic conditions.

In the thermophilic biogas plant analyzed in this study, isolates closely related to *Tepidanaerobacter syntrophicus* (16S rRNA identity 96.2–96.6 %) were found (Table 3). However, considerable differences in the 16S rRNA gene sequence indicate that the isolates do not belong to the species *Tepidanaerobacter syntrophicus* and therefore may possess different metabolic features. In addition, metagenome and metatranscriptome analysis revealed that members of the genus *Syntrophaceticus* are present in the community. As indicated by the much lower 16S rRNA abundance compared to *Tepidanaerobacter syntrophicus* (0.01 vs 1.14 %, Fig. 3), *Syntrophaceticus* spp. seem to possess lower metabolic activity in the analyzed system as deduced from metatranscriptome data. For both potentially syntrophic species resp. genera, corresponding methanogenic partners can be proposed. *Methanothermobacter* spp. and *Methanoculleus* sp. isolates representing such potential candidate partners were also cultivated (see Table 3).

Members of other genera comprising known SAOB were not found. However, for the class *Thermotogae*, *Defluviitoga tunisiensis* isolates were obtained. They feature high metabolic activity in the anaerobic digestion process as deduced from metatranscriptome sequence data (16S rRNA abundance 9.2 %). It may be speculated that this species also lives in a quasi syntrophic association with *Methanothermobacter* spp. or *Methanoculleus* sp. as it was shown, for example, for *Thermotoga lettingae* and *Methanothermobacter thermautotrophicus* [78].

Similar to SAOB, syntrophic propionate oxidizing bacteria (SPOB) were recognized to participate in anaerobic digestion and biomethanation in the literature [79]. SPOB were characterized within the class *δ*-*Proteobacteria*, for example, members of the genera *Smithella* and *Syntrophobacter*, as well as within the class *Clostridia*, for example members of the genera *Pelotomaculum* and *Desulfotomaculum*. Similar to SAOB, SPOB live in syntrophy with hydrogenotrophic methanogens, namely *Methanospirillum hungateii*, *Methanothermobacter thermautotrophicus*, or *Methanobacterium thermoautotrophicum* with optimal growth rates at mesophilic or thermophilic temperatures. Despite the absence of larger cell numbers of the genera *Methanospirillum* and *Methanobacterium* members in the analyzed thermophilic biogas plant, several hints for the presence of SPOB were found in the metagenomic datasets. Sequences were assigned to the genera *Pelotomaculum* (0.13 % of all metagenomic sequences), *Desulfotomaculum* (0.06 %), and *Syntrophobacter* (>0 %). However, the only isolate with a potential as SPOB is most closely related to *Desulfotomaculum australicum*, a species without reported syntrophy with methanogens.

### *The thermophilic biogas microbiome—the third stage, methanogenic* Archaea

For the generation of biogas rich in energy, namely in methane, the presence of methanogenic *Euryarchaeota* is indispensable. In mesophilic biogas plants digesting agriculture-derived substrates, i.e. manure from husbandry, 'energy crops', and/or residual wastes of agricultural production, the methane was predominantly produced by hydrogenotrophic *Methanomicrobiaceae* such as *Methanoculleus* spp. and/or *Methanospirillum* spp. [7, 13, 23, 41, 72, 80, 81]. In some cases, a predominance of acetoclastic methanogens, e.g., *Methanosaeta* sp. [6] or mixotrophic methanogens, e.g. *Methanosarcina* sp. [82] was observed.

In biogas plants operated under thermophilic conditions, a lower diversity within the methanogenic *Archaea* was found compared to mesophilic plants [6]. Typically, archaeal members of thermophilic methanogenic communities in production-scale plants are *Methanobacterium* sp., *Methanobrevibacter* sp., *Methanoculleus* sp., and *Methanothermobacter* sp., however, in varying abundance presumably depending on the prevailing abiotic fermentation conditions [9, 23, 83–85].

Generally, at thermophilic conditions, acetoclastic methanogens seem to be outcompeted by hydrogenotrophic methanogens [86]. This might also be the case in the thermophilic biogas plant analyzed in this study, where exclusively members of the hydrogenotrophic genera *Methanothermobacter* spp. and *Methanoculleus* sp. were found. This is in accordance with previously published studies analyzing laboratory-scale biogas fermenters under controlled conditions [86–88]. In addition, [87, 88] could show by automated fermentation with 'energy crops' as substrate that it was a reversible process between the dominance of *Methanothermobacter* spp. or *Methanoculleus* sp. depending only on temperature.

As outlined above, the 3- to 5-fold higher partial pressure of H_2_ under thermophilic conditions [35] could explain why hydrogenotrophic methanogens, e.g., *Methanomicrobiales* and *Methanobacteriales* but also *Methanosarcinales* dominate in an environment with a high substrate load. Members of the *Methanomicrobiales* exhibit a high affinity for H_2_, i.e. exhibit low threshold concentrations concerning their ability to utilize H_2_ as substrate, possibly providing an advantage over certain *Methanobacteriales* under conditions of low H_2_ partial pressure.

Interestingly, despite the higher cell abundance of rod-shaped *Methanothermobacter* spp., *Methanoculleus* species appeared to be highly metabolically active as deduced from metatranscriptome analysis. Fermentations conducted at laboratory scale revealed that certain abiotic process factors may support the succession of *Methanothermobacter* sp. by *Methanoculleus* sp. [89]. However, these experiments showed that both hydrogentrophic methanogens can be replaced by members of the mixotrophic methanogenic genus *Methanosarcina* [9, 18, 89] which was proposed as comprising the most versatile methanogenic genus [90]. In the thermophilic biogas plant analyzed in this study, the minor occurrence of *Methanosarcina* sp. was indicated only by DNA- and RNA-based analyses but not by microscopic analysis or cultivation (Figs. 2, 3). Hence, it can be assumed that the abundance and metabolic activity of *Methanosarcina* sp. in the analyzed biogas plant is poor compared with the other methanogenic species. However, the presence of *Methanosarcina* could have been overlooked by microscopy as, at high osmotic conditions, *Methanosarcina* (e.g., *M. mazei*) might exhibit non-typical coccoid single cells instead of clumps or aggregates [91]. Therefore, it still remains an unsolved question whether the thermophilic biomethanation process of agricultural substrates can be strengthened by promoting the growth of thermophilic *Methanosarcina* sp., for example, as previously isolated from thermophilic biogas plants [92].

In the analyzed thermophilic biogas plant, the microscopic analysis revealed 5.1 × 10^8^ methanogens per ml which is in accordance with earlier studies determining between 10^7^ and 10^8^ archaeal cells per ml in the fermenter fluid of agricultural biogas plants [7]. However, the abundance of methanogenic *Archaea* can range from 0.1 up to 20 % [86]. In biogas plants with a good performance, the methanogenic cell counts can reach more than 10^9^ per ml [23, 93]. In this biogas plant, methanogens accounted for 1.4 % of the total cell counts, mostly comprising rod-shaped *Methanothermobacter* spp., which is a comparatively low proportion. Usually the methanogenic counts of good operating thermophilic biogas plants lie in the range of 3–10 %. Nevertheless, the estimated performance of the biogas plant was excellent.

As indicated by analysis of the metatranscriptome, *Methanoculleus* sp. was the most active methanogen featuring a cell abundance of only 0.3 % of the total cell concentration. One reason for this finding might be that *Methanothermobacter* was more resistant to cell disruption for DNA and RNA preparation as described previously for this species [94], while the cell walls of *Methanoculleus* species are sensitive to lysis by detergents [95]. However, in this study, five different protocols for preparation of metagenomic DNA were applied to overcome such effects. A more reliable conclusion might be that at the prevailing conditions *Methanoculleus* was one of the ‘workhorses’ resp. key methanogens within this biogas plant.

### *The thermophilic biogas microbiome*—*human*-*pathogenic* Bacteria

Recently, there is an increasing discussion whether there is a risk regarding persistence or proliferation of human- and/or animal-pathogenic *Bacteria* species in biogas plants, namely concerning *Escherichia coli* and *Clostridium botulinum*, but also pathogenic species of the genera *Salmonella, Listeria, Campylobacter,Enterococcus*, and others. Previous studies showed that, beside other factors, an increase in the operation temperature of biogas plants is predominantly effective to reduce the amount of viable pathogens [20, 96, 97]. Hence, it can be assumed that, despite the supply of cattle and pig manure, in the biogas plant analyzed in this study operated under thermophilic conditions (54 °C), pathogens are detectable only when cultivated at lower temperature levels around 37 °C.

By conventional cultivation under anoxic or microoxic conditions and subsequent MALDI-TOF MS-based identification, pathogenic species (according to German risk group 2) were detected in the fermenter of the biogas plant in amounts of up to 10^7^ CFU g^−1^ in case of *Bacillus cereus* and 10^4^ CFU g^−1^ in case of *Clostridium perfringens* and *Clostridium sporogenes* (Additional file 1: Figure S4). In contrast, prominent pathogenic members of the class *γ*-*Proteobacteria* such as *Escherichia coli* or *Salmonella enterica* were not found in the fermenter liquid sample by cultivation.

With the exception of *E. coli* and *C. perfringens*, none of the pathogens detected in the manure substrate were found in the digestate after fermentation. However, it must be noticed that for unknown reasons some other pathogens were cultivated which were neither detected in the manure substrate nor in the fermenter liquid such as pathogenic species from the genera *Morganella*, *Proteu*s and *Salmonella*.

### Definition of the core microbiome by applying culturomics—towards a microbial resource management for thermophilic biogas plants

With respect to the certainly existing, but up to now widely unexploited potential of thermophilic anaerobic digestion and biomethanation, there is an obvious need for a better understanding of the structure, the dynamics, and the metabolic capacity of microbial communities involved in this bioprocess. Microbial resource management (MRM) strategies as proposed by [98] are crucial for the evaluation and subsequent optimization of biotechnologically used microbial trophic networks. An essential part of MRM and indispensable pre-requisite for further biotechnological engineering is to link the community organization to its functioning. This can be achieved by advanced molecular analyses such as studies of the microbial metagenomes and metatranscriptomes, in combination with traditional microbiological studies, namely isolation and characterization of microbial strains.

However, the interpretation of molecular data derived from massive sequencing by bioinformatical methods is just as good as the available reference data. In this study, only 18–25 % of 16S rRNA gene or gene-transcript sequences could be classified at genus level indicating both, the presence and activity of a huge majority of currently unknown microorganisms. The same was found for other genes, e.g. encoding enzymes responsible for metabolic features, by analysis of the microbial metagenome. In this context, it must be noticed that the comparatively high assignment for non-16S-rRNA sequences found in the microbial metatranscriptome was caused by 5S and 23 rRNA species but only in much lower abundance for mRNA. To unravel the most active metabolic pathways of microorganisms in a thermophilic biogas plant, the analysis of a 16S rRNA depleted microbial transcriptome seems to be indispensable.

Examples for new microbial species or genera participating in the anaerobic digestion process within biogas plants were recently published, e.g. by [35, 74, 99]. The value of new genome data for the interpretation of metagenome and metatranscriptome was proven in this study considering as examples *Herbinix hemicellulosilytica* and *Defluviitoga tunisiensis* underlining the up to now only poorly recognized role of *D. tunisiensis* in thermophilic anaerobic digestion of agricultural residues. Hence, for understanding the complex trophic networks present in biogas plants, but also in other biotechnological systems, it seems to be indispensable to apply culturomics as proposed by [100] in a study of the human gut microbiome. Besides the creation of new and basic microbiological knowledge, i.e. by the description of new taxonomic units at different levels, knowledge about microbial ecology and population dynamics will be gained which is indispensable for the establishment of MRM strategies and the subsequent knowledge-based optimization of technical solutions for the biomethanation of biomass.

However, 95–99.9 % of the microorganisms are not readily culturable by standard laboratory techniques [101]. Furthermore, traditional isolation methods are labor-intensive and time-consuming, due to the need for serial enrichment cultures and the slow growth of microorganisms under suboptimal conditions. Hence, new advanced molecular approaches such as single-cell sequencing provide a good possibility making the genomic information of individual cells accessible without the complications of culturing them. Unfortunately, up to now such approaches are difficult to apply, e.g. due to the limitations of automatic sorting systems.

Recently, culture-independent molecular techniques such as differential coverage binning, taxonomic binning and assembly allow draft genome reconstructions of community members for which sequencing has recovered substantial amounts of sequences [102]. As example, this approach was successfully applied to obtain draft genomes from two microbial populations previously identified in an industrial wastewater treatment bioreactor [103]. Applied binning will allow metabolic reconstruction and therefore the prediction of a microorganism’s role in the corresponding communities.

## Conclusions

In this study, a first step towards the definition of a functional core microbiome for the anaerobic digestion and biomethanation of 'energy crops' together with manure from husbandry at thermophilic temperature regime was taken. Several important insights into the complex biogas microbiome were gained in this study, which provide a solid basis for more detailed analysis of the microbial systems ecology and its further biotechnological optimization.

By a polyphasic approach combining classical cultivation and physiological resp. molecular characterization of the obtained isolates complemented by metagenome and metatranscriptome community analyses, members of the genera *Defluviitoga*, *Clostridium* cluster III, and *Tepidanaerobacter* were determined as most metabolically active fermentative *Bacteria* in an exemplary sampled industrial-scale thermophilic biogas plant converting maize and barley silage in co-digestion with cattle and pig manure. As methanogenic *Archaea*, members of the genus *Methanoculleus* were found to be most active.

However, it should be noticed that this study also revealed a huge number of up to now unknown or insufficiently characterized *Bacteria* species existing in thermophilic biogas plants featuring unknown functions in anaerobic digestion and biomethanation. This indicates the absolute necessity for further micro- and molecular biological research for a better understanding of biomethanation processes in industrial-scale biogas plants.

## Additional file

10.1186/s13068-016-0581-3 Additional information is available at *Biotechnology for Biofuels* journal’s website as pdf document, Maus_et_al_v2016-08-03_additional_file_1.pdf. This file contains Additional information on methods as well as additional figures and tables as follows: (i) Additional information on methods for extraction of total microbial RNA. (ii) Additional information on methods for identification of isolates. (iii) **Table S1.** Statistics of quality-controlled metagenomes sequences data for the microbial community of the thermophilic production-scale biogas plant. (iv) **Table S2.** Statistics of taxonomically classified sequences obtained from the metagenome and metatranscriptome datasets. (v) **Table S3.** Number of 16S rRNA sequences and corresponding percentages of bacterial and archaeal genera within the analyzed metagenome and metatranscriptome datasets. The number of metatranscriptome-derived 16S rRNA sequences, i.e. 532,381, was normalized to the number of metagenome-derived sequences, i.e. 18,817. Only genera with at least 10 (normalized) sequences in one of the datasets were considered. ND = no sequences were determined in the dataset. (vi) **Figure S1.** Exemplary work-flow illustration of the different strategies for the isolation of cellulolytic bacteria. Isolation strategy (3): direct enrichment and isolation using different media. Isolation strategy (4): serial dilution (i.e. dilution to extinction) in liquid medium. Isolation strategy (5): direct plating on solid medium without previous enrichment in liquid culture. (vii) **Figure S2.** Relative abundance of the 25 most abundant families within the biogas microbial community of the thermophilic biogas plant as deduced from 16S rRNA gene sequences of combined metagenome datasets (>= 0.05 % of the sequences in the metagenome). Taxonomic assignments of the 16S rRNA gene sequences were obtained applying BLASTN against the RDP database applying MGX. (viii) **Figure S3.** Relative abundance of the 25 most abundant families within the biogas microbial community of the thermophilic biogas plant as deduced from 16S rRNA sequences of the metatranscriptome datasets (>= 0.05 % of the sequences in the metatranscriptome). Taxonomic assignments of the 16S rRNA sequences were obtained applying BLASTN against the RDP database applying MGX. (ix) **Figure S4.** Cultivable microbial species applying the isolation strategies (1)–(2) and corresponding cell counts in samples of substrate (swine manure), fermenter content (main fermenter), and digestate (second fermenter). The cultivation temperature was 37 or 50 °C (*), respectively. (x) **Figure S5.** Light- (a) and fluorescent (b) microscopic picture of *Methanothermobacter marburgensis* isolated from the thermophilic biogas plant. (xi) **Figure S6.** Phylogenetic tree of selected archaeal (a) and bacterial (b) isolates in relation to corresponding type species. The 16S rRNA gene sequences from closely related type species were obtained from the SILVA database [47]. The isolate T3/55^T^ represents the type strain for a novel genus and species, namely *Herbinix hemicellulosilytica*, which was recently described by [35]. (xii) **Figure S7.** Fragment recruitment of metagenome sequences derived from the microbial community of the analyzed thermophilic biogas plant to the genomes of the strains *Clostridium cellulosi* str. DG5 (a), *Herbinix hemicellulosilytica* str. T3/55^T^ (b) and *Defluviitoga tunisiensis* str. L3 (c). Only metagenome sequence reads with more than 75 % sequence identity (y-axis) to the corresponding genome were mapped. Percent identity (y-axis) of a mapped metagenome read was plotted against the mapping position on the genome sequence (x-axis) of *H. hemicellulosilytica* str. T3/55^T^ or *D. tunisiensis* str. L3. The x-axis represents the extension (scale) of the *C. cellulosi* str. DG5, *H. hemicellulosilytica* str. T3/55^T^ or *D. tunisiensis* str. L3 genome. Sequence coverage is visualized by gray scale intensity (right margin). Matching sequence read length is indicated by the diameter of the circle representing the mapping position (scale is given on the top right).

## Abbreviations

CAZy: carbohydrate-active enzyme database
CBM: carbohydrate binding module families
CFU: colony forming units
COG: clusters of orthologous groups of proteins
conc.: concentration
DM: dry matter
FID: flame ionization detector
FM: fresh mass
GH: glycosyl hydrolase
PASC: phosphoric acid swollen cellulose
QMF: quantitative microscopic fingerprinting
RDP: ribosomal database project
SAOB: syntrophic acetate oxidizing bacteria
SPOB: syntrophic propionate oxidizing bacteria
VFA: volatile fatty acids
VS: volatile solids
