# Supplementary material for: Unraveling the microbiome of a thermophilic biogas plant by metagenome and metatranscriptome analysis complemented by characterization of bacterial and archaeal isolates
Source: Biotechnol Biofuels. 2016 Aug 11;9:171. doi: 10.1186/s13068-016-0581-3 (PMC4982221; doi:10.1186/s13068-016-0581-3)
Supplement: Supplementary file 1 — 10.1186/s13068-016-0581-3 Additional information is available at Biotechnology for Biofuels journal’s website as pdf document, Maus_et_al_v2016-08-03_additional_file_1.pdf. This file contains Additional information on methods as well as additional figures and tables as follows: (i) Additional information on methods for extraction of total microbial RNA. (ii) Additional information on methods for identification of isolates. (iii) Table S1. Statistics of quality-controlled metagenomes sequences data for the microbial community of the thermophilic production-scale biogas plant. (iv) Table S2. Statistics of taxonomically classified sequences obtained from the metagenome and metatranscriptome datasets. (v) Table S3. Number of 16S rRNA sequences and corresponding percentages of bacterial and archaeal genera within the analyzed metagenome and metatranscriptome datasets. The number of metatranscriptome-derived 16S rRNA sequences, i.e. 532,381, was normalized to the number of metagenome-derived sequences, i.e. 18,817. Only genera with at least 10 (normalized) sequences in one of the datasets were considered. ND = no sequences were determined in the dataset. (vi) Figure S1. Exemplary work-flow illustration of the different strategies for the isolation of cellulolytic bacteria. Isolation strategy (3): direct enrichment and isolation using different media. Isolation strategy (4): serial dilution (i.e. dilution to extinction) in liquid medium. Isolation strategy (5): direct plating on solid medium without previous enrichment in liquid culture. (vii) Figure S2. Relative abundance of the 25 most abundant families within the biogas microbial community of the thermophilic biogas plant as deduced from 16S rRNA gene sequences of combined metagenome datasets (>= 0.05 % of the sequences in the metagenome). Taxonomic assignments of the 16S rRNA gene sequences were obtained applying BLASTN against the RDP database applying MGX. (viii) Figure S3. Relative abundance of the 25 most abundant [file 13068_2016_581_MOESM1_ESM.pdf]

Unraveling the microbiome of a thermophilic biogas plant by metagenome and meta-transcriptome analysis complemented by characterization of bacterial and archaeal isolates

- Additional file 1 -

Irena Maus[1] (irena.maus@cebitec.uni-bielefeld.de), Daniela E. Koeck[2] (daniela.koeck@mytum.de), Katharina G. Cibis[3] (katharinacibis@gmx.de), Sarah Hahnke[4] (shahnke@atb-potsdam.de), Yong S. Kim[5] (yongsung.kim@haw-hamburg.de), Thomas Langer[4] (tlanger@atb-potsdam.de), Jana Kreubel[3] (kreubel@uni-mainz.de), Marcel Erhard[6] (merhard@ripac-labor.de), Andreas Bremges[1,7] (andreas@cebitec.uni-bielefeld.de), Sandra Off[5] (sandra.off@haw-hamburg.de), Yvonne Stolze[1] (ystolze@cebitec.uni-bielefeld.de), Sebastian Jaenicke[8] (sebastian.jaenicke@computational.bio.uni-giessen.de), Alexander Goesmann[8] (alexander.goesmann@computational.bio.uni-giessen.de), Alexander Sczyrba[1,7] (asczyrba@techfak.uni-bielefeld.de), Paul Scherer[5] (paul.scherer@haw-hamburg.de), Helmut König[3] (hkoenig@uni-mainz.de), Wolfgang H. Schwarz[2] (wschwarz@wzw.tum.de), Vladimir V. Zverlov[2] (zverlov@hotmail.com), Wolfgang Liebl[2] (wliebl@wzw.tum.de), Alfred Pühler[1] (puehler@cebitec.uni-bielefeld.de), Andreas Schlüter[1] (aschluet@cebitec.uni-bielefeld.de), Michael Klocke[4*] (mklocke@atb-potsdam.de)

[1] Bielefeld University, Center for Biotechnology (CeBiTec), Institute for Genome Research and Systems Biology, Universitätsstr. 27, 33615 Bielefeld, Germany

[2] Technische Universität München, Department of Microbiology, Emil-Ramann-Str. 4, 85354 Freising-Weihenstephan, Germany

[3] Johannes Gutenberg-University, Institute of Microbiology and Wine Research, Becherweg 15, 55128 Mainz, Germany

[4] Leibniz-Institut für Agrartechnik Potsdam-Bornim e.V. (ATB), Dept. Bioengineering, Max-Eyth-Allee 100, 14469 Potsdam, Germany

[5] University of Applied Sciences Hamburg (HAW), Faculty Life Sciences / Research Center 'Biomass Utilization Hamburg', Ulmenliet 20, 21033 Hamburg-Bergedorf, Germany

[6] RIPAC-LABOR GmbH, Am Mühlenberg 11, 14476 Potsdam-Golm, Germany

[7] Bielefeld University, Faculty of Technology, Universitätsstr. 25, 33615 Bielefeld, Germany

[8] Justus-Liebig University Gießen, Department of Bioinformatics and Systems Biology, Heinrich-Buff-Ring 58, 35392 Giessen, Germany

*Corresponding author

Dr. Michael Klocke

Leibniz-Institut für Agrartechnik Potsdam-Bornim e.V., Max-Eyth-Allee 100, 14469 Potsdam, Germany, email: mklocke@atb-potsdam.de

Methods for extraction of total microbial RNA

In order to extract the total microbial RNA, the protocol previously described by [19] was applied. Briefly, the following steps were carried out: 4.5 g fermenter sample and 2.5 ml TE-buffer (4 °C) were applied on a falcon filter (pore size 40 µm; BD, Belgium) and spun at 4000 x g for 2 min. Equal volumes (1 ml) of acid phenol (4 °C) and of glass beads (diameter 0.1 mm) were added to the filtrate and vortexed two times at maximum speed for 4 min, interrupted by cooling on ice for 1 min in between. The mixture was then centrifuged at 5500 x g for 5 min; and the upper phase used for the further processing with the Qiagen RNeasy Midi kit according to manufacturer's instructions. Finally, the RNA was eluted in 150 µl RNAse-free water.

The remaining DNA within the RNA sample was removed twice using DNase I (Roche) as well as the DNase and the RNeasy Mini kit (Qiagen). In short, the following steps were performed: 80 µl RNA solution were mixed with 10 µl DNase I and 10 µl DNase buffer (Roche) and incubated for 1.5 h at room temperature under constant shaking at 400 rpm. Subsequently, 350 µl supplied RTL buffer and 250 µl ethanol (100 %) were added; and the mixture was applied on the supplied columns. After centrifugation (13000 rpm, 30 sec), 250 µl RW1 buffer (Qiagen) were applied on the column and centrifuged again. Subsequently 10 µl DNase and 70 µl DNase RDD buffer (Roche) were added on the column membrane and incubated for 15 min at room temperature, followed by another washing step with RW1 buffer. Two additional washing steps were carried out with 500 µl RPE buffer (Qiagen) and the membrane was dried by centifugation (13000 x g, 2 min) followed by RNA eluation in 40 µl RNase-free water (13000 x g, 1 min).

The successful treatment with DNase was verified by PCR applying the universal primer pair Arch-0519-a-S-15 / S-D-Bact-0785-b-A-18 (5' → 3': CAGCMGCCGCGGTAATWC / TACNVGGGTATCTAATCC) targeting the 16S rRNA gene [104]. Concentrations of the DNA-free RNA samples were determined using the RNA 6000 Pico Chip kit (Agilent, Waldbronn, Germany).

Methods for isolation of *Bacteria* and *Archaea*

Isolation strategy (1) targeting mesophilic pathogenic Bacteria*:* The samples were analyzed with qualitative and quantitative methods under microoxic and anoxic culture conditions at 37 °C growth temperature. From each sample, a serial dilution up to 10^{-9} was prepared in buffered peptone water. 100 µl of each dilution were plated four times on Columbia Blood Agar (CSB), Columbia Blood Agar with Neomycin (NEO), Columbia Blood Agar with Gentamycin (GENTA), Plate Count AGAR (PC), Polymyxin Egg Yolk Mannitol Bromothymol blue Agar (PEMBA), and Sabouraud Dextrose Agar with Chloramphenicol (SABC). One set of plates, *i.e.* duplicates, was cultivated for 48 h under microoxic conditions. The other set of plates, for (facultative) anaerobic bacteria, was cultivated under anoxic conditions for 48 h. The anaerobic culture conditions were generated using the AnaeroGen 2.5l (Atmosphere Generation System, Thermo Scientific, Oxoid Basingstoke, UK). All plates with colonies in the countable range from the same dilution were analyzed. All colonies with similar morphology were counted and identified using the MALDI-TOF MS method. Therefore, the MALDI-TOF MS sample preparation was performed similar to [31].

Isolation strategy (2) targeting thermophilic pathogenic Bacteria*:* Similar to strategy (1), with the following modification: All samples were cultivated at 50 °C.

Isolation strategy (3) targeting cellulolytic Bacteria*:* For cultivation of cellulolytic microorganisms, GS2 medium [32] or mineral medium [33] were used under anoxic conditions (Supplementary Figure S1). The media were supplemented with 0.5 % (w/v) Avicel in liquid cultures and 0.5 % (w/v) phosphoric acid swollen cellulose (PASC) [34] or Avicel on solid agar plates to enrich for cellulolytic bacteria. Additionally, clarified cattle rumen content or digestate from a biogas plant was added either before autoclaving or sterile filtrated in different concentrations to support growth. A total of 1 ml of the sludge was suspended in 50 ml NaCl solution (0.2 M) and freshly inoculated into autoclaved medium. The culture was incubated at 55 °C until filter paper stripes (Whatman No. 1) showed obvious degradation and again transferred into the same medium. After four to six repetitions the resultant enriched culture was serially diluted up to 10^{-4}. An aliquot (0.1 ml) of the dilutions was plated on agar plates (2 % agar) in an anaerobic chamber (95 % N_2 + 5 % H_2; Coy Laboratory Products) and overlaid with GS2 Agar (2 % w/v) containing 0.5 % (w/v) Avicel or PASC. The agar plates were incubated anoxically at 55 °C. Colonies with different morphology were streaked to single colonies three times. Single colonies were picked and inoculated to the same medium with 0.5 % (w/v) filter paper to confirm cellulose degradation and purity.

Isolation strategy (4) targeting cellulolytic Bacteria*:* Isolation strategy (4) is comparable to the strategy described by [35], with some modifications. Briefly, 1 ml digester sludge was suspended in 50 ml NaCl solution (0.2 M) and serially diluted to 10^{-8}. Each dilution was freshly inoculated into GS2 medium with 0.5 % (w/v) filter paper (Whatman No. 1) as sole carbon source and 0.5 % (v/v) rumen extract to enrich cellulolytic microbes. The culture was incubated at 55 °C under anoxic conditions until the filter paper substrate showed obvious degradation. The highest dilution with filter paper degradation was diluted to 10^{-8} and transferred into the same medium. After four repetitions of dilution and growth in fresh medium, the resultant enriched culture was serially diluted up to 10^{-4}. Cultivation on solid medium and isolation was performed as described for isolation strategy (3).

Isolation strategy (5) targeting cellulolytic Bacteria*:* Similar to strategy (3), with the following modifications: the dilution of the suspended sludge was directly plated on agar plates containing 0.05 % (w/v) of cellobiose as carbon source and overlaid with GS2 Agar (2 % w/v) containing 0.5 % (w/v) Avicel or PASC. Colonies producing clear halos due to cellulolytic activity were streaked to single colonies three times under anoxic conditions.

Isolation strategy (6) targeting acidogenic/acetogenic Bacteria*:* Acidogenic bacteria were isolated with emphasis on acetic acid, propionic acid and butyric acid forming bacteria. For the isolation from the thermophilic biogas plant, a minimal medium, *i.e.* modified DSMZ medium 287 [36] was used, which was supplemented with one carbon source (6 g l^{-1} of Na^{+}-DL-lactate, succinate, glucose or a mixture of the following six amino acids: L-alanine, L-serine, L-threonine, L-cystein, L-glutamic acid, and L-methionine). These substrates are potential primary fermentation products of anaerobic hydrolysis of polymeric biomass, which can be converted by microorganisms to acetic acid, propionic acid, or butyric acid. Detailed information about the isolation and identification of acid forming bacteria are described by [37]. Anoxic procedures such as preparing of nutrition media and isolation of acid forming bacteria were performed in an anaerobic chamber (Coy Laboratory Products, USA). Firstly, acidogenic bacteria were enriched by the addition of 500 mg of reactor sample to 9 ml minimal medium containing the corresponding carbon source. The incubation was performed at 54 °C. To obtain pure cultures, the deep agar shake method was applied for the mentioned nutrition media. Formation of acetic acid, propionic acid and butyric acid in bacterial cultures was analyzed by high performance liquid chromatography (HPLC, Shimadzu, Kyoto, Japan) with the column Hpx87 (size: 300 x 7.8 mm, 9 µm particle size; BioRad, Germany) and the RI-Detector RID-10A (Shimadzu, Germany).

Isolation strategy (7) targeting acidogenic/acetogenic Bacteria*:* Similar to strategy (6), with the following modification: instead of the deep agar shake method, plating was performed on the above mentioned, anoxic nutrition media to obtain pure cultures of acid producing bacteria.

Isolation strategy (8) targeting different anaerobic thermophilic Bacteria*:* To isolate a variety of different bacteria, anoxic media with different substrates were used: (a) R2A medium, which contained complex substrates such as yeast extract, peptone and casamino acids as well as glucose, starch and pyruvate, was prepared according to [38] with addition of (l^{-1}) 0.75 g $MgCl_2$ * 6 H_2O, 0.5 g cysteine-HCl * H_2O and 1 mg resazurin; pH was adjusted to 7.2. (b) Nitrate broth medium, containing the substrates peptone, acetate, ethanol and propionate, was prepared as described by [39]. It was supplemented with (l^{-1}) 0.5 g cysteine-HCl * H_2O and 1 mg resazurin. The pH value was adjusted to 7.0. (c) The minimal medium as described by [38] was prepared with 5.35 g NH_4NO_3 and 0.42 g $NaNO_3$ (l^{-1}) and supplemented with 0.68 g sodium formate, 1.8 g glucose, 0.5 g cysteine-HCl * H_2O and 1 mg resazurin. The pH value was adjusted to 7.1. (d) The BM/NO^{3-} medium, containing succinate as sole substrate, was prepared according to [40] and supplemented with (l^{-1}) 0.5 g cysteine-HCl * H_2O and 1 mg resazurin. The pH value was adjusted to 7.5. (e) The DSMZ medium 287 [36] was modified according to [41] and supplemented with 1 mg l^{-1} Na_2WO_4*H_2O, sodium acetate and formate (10 mM each), 0.5 % methanol and 5 % reactor sludge.

Sludge was prepared as described for DSMZ medium 119 [105]. Cultivation was performed on solid and in liquid media. All solid media contained 15 g l^{-1} agar, liquid media were prepared under anoxic conditions, filled in hungate tubes and autoclaved under N_2 gas atmosphere (80 % H_2 and 20 % CO_2 gas mixture in case of DSMZ medium 287). For isolation of bacteria the reactor sample was diluted 10^1 to 10^7 fold in the media (a) - (d). Aliquots of the dilutions were spread on pre-reduced agar plates of the respective medium and incubated anoxically at 50 °C. Single colonies were picked and re-streaked several times for purification. The liquid assays were incubated for two to four weeks at 50 °C. Afterwards aliquots of the cultures were transferred to fresh medium. After the seventh transfer, 100 µl of enrichment culture were spread on BBL™ Columbia Agar Base medium (Th. Geyer, Germany) supplemented with 5 % laked horse blood (Oxoid, Germany). Agar plates were incubated anaerobically at 50 °C; and colonies were re-streaked for purification. Medium (e) was only used for liquid cultures. The medium was inoculated in triplicates with 50 µl reactor material and incubation occurred at 50 °C. After four weeks of incubation, aliquots of the cultures were transferred into the same medium supplemented with two antibiotics (100 µg ml^{-1} vancomycin * HCl and 60 µg ml^{-1} streptomycin). After

incubation of four weeks, the cultures were transferred into medium containing another combination of antibiotics (100 µg ml^{-1} sodium ampicillin and 60 µg ml^{-1} kanamycin) and after another incubation period fresh medium with vancomycin * HCl and sodium ampicillin (100 µg ml^{-1} each) was used. Incubation with different combinations of antibiotics was repeated three times. Afterwards, isolation of bacteria occurred on BBL™ Columbia Agar Base medium supplemented with 5 % horse blood as described above.

Isolation strategy (9) targeting facultative anaerobic thermophilic Bacteria*:* To isolate facultative anaerobic bacteria 10 % DEV nutrient agar (Merck, Germany) was used. Therefore, 10 % of the recommended amount of the medium was used per liter and 4.5 g NaCl and 16.2 g agar (l^{-1}) were added to adjust the salt and agar concentrations to that of the non-reduced medium. The reactor sample was diluted 10^4 and 10^6 fold, plated on 10 % DEV nutrient agar plates and incubated under exposure to air oxygen at 50 °C. Single colonies were picked and re-streaked for purification.

Isolation strategy (10) targeting methanogenic Archaea*:* A cultivation technique for strictly anaerobic microorganisms was performed in accordance to the recommendations by [42], but without usage of an anaerobic chamber. H_2 (80 %) and CO_2 (20 %) were fed to the gas phase of cultivation vials with up to 2 bar overpressure. For cultivation of hydrogenotrophic methanogens, a medium containing following components were used: 10 mM imidazole buffer adjusted with HCl to 7.0, 5 mM $MgCl_2$ * 6 H_2O, 30 mM NaCl, 18.6 mM NH_4Cl, 0.5 mM $CaCl_2$ * 2 H_2O, 64.1 mM KCl, 1 mM sodium polyphosphate $(NaPO_3)_n$ (chain length n about 100). 0.5 µM $ZnCl_2$, 0.1 µM $MnCl_2$ * 4 H_2O, 0.1 µM H_3BO_3, 0.1 µM $CuCl_2$ * 2 H_2O, 0.1 µM $NaWO_4$ * 2 H_2O, 0.5 µM $CoCl_2$ * 6 H_2O, 0.5 µM $NiCl_2$ * 6 H_2O, 0.1 µM Na_2SeO_3 * 5 H_2O, 0.1 µM Na_2MoO_4 * 2 H_2O, 10 µM $Fe(III)Cl_3$ * 6 H_2O, and vitamin solution according to [106]. 5 mM $NaHCO_3$, 5 mM sodium acetate, 10 mM sodium formate, 8 µM resazurin, 0.43 mM cysteine, 1 mM Na_2S and 0.083 mM titan citrate adjusted to pH 7.0. The reducing agents cysteine, Na_2S and titan citrate were finally added as separate solutions. For the isolation of target methanogens in liquid medium, dilution series and repetition of transfer to fresh medium were conducted. During this procedure, the most dominant methanogens remained in the culture. The culture with the added antibiotics was first incubated at 37 °C for one or two days to let the antibiotics work before moving to the selective cultivating temperature, *e.g.* 53 °C. In the first dilution step, a combination of five antibiotics was used, *i.e.* 20 µg ml^{-1} cefoxitin, 4 µg ml^{-1} cefsulodin, 20 µg ml^{-1} vancomycin, 20 µg ml^{-1} bacitracin, and 50 µg ml^{-1} ampicillin, together with fresh medium. After the third dilution step the antibiotics bacitracin and ampicillin were omitted and after the fifth transfer only vancomycin was applied as antibiotic, mainly to sustain the homogeneity of the apparently pure culture. Further details on the cultivation procedures were previously published by [107]. Growth and purity of the cultures were confirmed microscopically and by molecular identification.

Isolation strategy (11) targeting methanogenic Archaea*:* The techniques for cultivating strict anaerobes described by [42] were used throughout the study. At first colonies picked from deep agar shake were incubated at 55 °C and then moved to the selective cultivating temperature, *e.g.* 65 °C. A combination of the antibiotics ampicillin and vancomycin (final concentration 100 µg ml^{-1}) was used. The deep agar shake method was performed in medium DSMZ 287 [36] supplemented with an amino acid solution (l^{-1}) 1.115 g L-arginine-HCl, 1.875 g DL-asparagine, 0.755 g L-cysteine, 0.915 g L-glutamate, 0.915 g L-glutamine, 1.01 g L-histidine-HCl, 0.82 g L-leucine, 1.14 g L-lysine-HCl, 0.93 g L-methionine, 0.82 g L-isoleucine, 1.03 g L-phenylalanine, 0.72 g L-proline, 0.655 g L-serine, 1.5 g DL-threonine, 1.28 g L-tryptophane, 1.18 g L-tyrosine, 1.46 g L-valine) in 1 : 1000 (v/v) dilution.

Methods for identification of isolates

Genomic DNA of the bacteria isolated with strategies (3), (4), and (5) was extracted from the culture using a bacterial genomic DNA kit (Molzym, Germany) according to manufacturer's guidelines. The purity of each isolate was examined by PCR of the 16S rRNA gene with the bacteria-specific oligonucleotide primers 616F and 630R, as described by [108]. PCR amplification and sequencing of the 16S rRNA genes were carried out as described previously [65]. Genomic DNA of the *Bacteria* and *Archaea* isolated with strategies (6), (7), and (11) was extracted using the DNeasy blood and tissue kit (Qiagen, Germany) according to the protocol for gram positive *Bacteria*. Thereafter, 16S rRNA genes were amplified with the primer set PurEubak5 and PurEubak3 for *Bacteria* [37], or with the primer set Met86f and Met1340r for methanogenic *Archaea* [41], and cleaned up with USB® ExoSAP-IT® PCR product cleanup kit (Affymetrix, USA). Subsequently, the PCR products were sequenced (LGC Genomics, Germany). To sequence the 16S rRNA genes of the isolates obtained with strategies (8) and (9), colonies of the isolates were suspended in 50 µl molecular biological grade nuclease-free water and cells were cracked by consecutive freezing and thawing three times. Amplification and subsequent sequencing of the almost complete 16S rRNA gene of the strains was carried out using the primer pair 27F and 1492R as described by [99]. For identification of the *Archaea* isolated with strategy (10), genomic DNA was extracted from 1 - 2 ml of liquid culture using the Gene Matrix stool DNA purification kit (Roboklon, Germany). The archaeal 16S rRNA gene fragment was sequenced by GATC Biotech (Germany) using the primers w017 (F) and w002 (R) [109].

Additional references

104. Klindworth A, Pruesse E, Schweer T, Peplies J, Quast C, Horn M, Glöckner FO. Evaluation of general 16S ribosomal RNA gene PCR primers for classical and next-generation sequencing-based diversity studies. Nucleic Acids Res. 2013;41:e1.

105. DSMZ medium 119. https://www.dsmz.de/microorganisms/medium/pdf/DSMZ_Medium119.pdf. Accessed 04 May 2016.

106. Wolin EA, Wolin MJ, Wolfe RS. Formation of methane by bacterial extracts. J Biol Chem. 1963;238:2882–6.

107. Scherer PA. Vanadium and molybdenum requirement for the fixation of molecular nitrogen by two Methanosarcina strains. Arch Microbiol. 1989;151:44–8.

108. Loy A, Schulz C, Lucker S, Schopfer-Wendels A, Stoecker K, Baranyi C, Lehner A, et al. 16S rRNA gene-based oligonucleotide microarray for environmental monitoring of the beta-proteobacterial order “Rhodocyclales”. Appl Environ Microbiol. 2005;71:1373–86.

109. Huang LN, Zhou H, Chen YQ, Luo S, Lan CY, Qu LH. Diversity and structure of the archaeal community in the leachate of a full-scale recirculating landfill as examined by direct 16S rRNA gene sequence retrieval. FEMS Microbiol Lett. 2002;214:235–40.

Table S1: Statistics of quality-controlled metagenomes sequences data for the microbial community of the thermophilic production-scale biogas plant.

Protocol used for microbial DNA extraction	Number of obtained DNA sequences (*i.e.* reads)	Total sequence data [bp]	Q30[6] value of sequenced bases [%]	Number of merged paired-end reads
**A[1]**	4054984	1058350824	88.71	1777661
**B[2]**	5521480	1441106280	89.62	2689150
**C[3]**	5975856	1559698416	89.34	2890795
**D[4]**	3933006	1026514566	89.10	1793876
**E[5]**	2803170	731627370	89.92	1323234

[1] A: FastDNA Spin Kit for Soil (MP Biomedicals, France)

[2] B: NucleoSpin Soil (Macherey-Nagel, Germany)

[3] C: PowerLyzer DNA Isolation Kit (MoBio, USA)

[4] D: PowerSoil DNA Isolation Kit (MoBio, USA)

[5] E: Classical chloroform-isoamyl alcohol DNA extraction [8]

[6] Probability of an incorrect base call 1 in 1000 times

Table S2: Statistics of taxonomically classified sequences obtained from the metagenome and metatranscriptome datasets.

Taxonomic affiliation	Metagenomic dataset				Metatranscriptomic dataset			
	All sequences[1]		16S rRNA gene sequences[2]		All sequences[1, 3]		16S rRNA sequences[2]	
	Number of sequences [-]	Relative abundance [%]	Number of sequences [-]	Relative abundance [%]	Number of sequences [-]	Relative abundance [%]	Number of sequences [-]	Relative abundance [%]
**Total sequences**	6500000	100	21888	100	1149525	100	547373	100
**Domain**	1162586	18	18817	86	793815	69	532381	97
**Phylum**	1012738	16	10271	47	731560	64	339680	62
**Class**	978234	15	7019	32	706392	61	247415	45
**Order**	953369	15	6099	28	705086	61	223389	41
**Family[4]**	910747	14	4985	18	693057	60	187071	34
**Genus**	910844	14	3842	18	685588	60	134563	25

[1] Taxonomic composition was determined by means of lowest common ancestor (LCA) algorithm implemented in MGX applying BLASTN against NCBI database.

[2] Taxonomic assignment were obtained applying BLASTN against RDP database implemented in MGX.

[3] Including 16S rRNA, 5S rRNA, 23S rRNA, mRNA, and other RNA species

[4] Currently some reference genera have no exact assignment at family level leading to their underrepresentation in the number of taxonomically identified sequences.

Table S3: Number of 16S rRNA sequences and corresponding percentages of bacterial and archaeal genera within the analyzed metagenome and metatranscriptome datasets. The number of metatranscriptome-derived 16S rRNA sequences, *i.e.* 532381, was normalized to the number of metagenome-derived sequences, *i.e.* 18817. Only genera with at least 10 (normalized) sequences in one of the datasets were considered. ND = no sequences were determined in the dataset.

Taxonomic classification						Metagenome[1]		Metatranscriptome[2]	
**Domain**	**Phylum**	**Class**	**Order**	**Family**	**Genus**	**Number of sequences [-]**	**Relative abundance [%]**	**Number of sequences [-]**	**Relative abundance [%]**
Archaea	*Euryarchaeota*	*Methanobacteria*	*Methanobacteriales*	*Methanobacteriaceae*	*Methanobacterium*	97	0.52	ND	
					Methanothermobacter	154	0.82	5	0.02
		Methanomicrobia	*Methanomicrobiales*	*Methanomicrobiaceae*	*Methanogenium*	22	0.12	3	0.01
					Methanosarcina	69	0.37	ND	
					Methanoculleus	519	2.76	1067	5.67
Bacteria	*Firmicutes*	*Bacilli*	*Lactobacillales*	*Lactobacillaceae*	*Lactobacillus*	19	0.10	ND	
		Clostridia	*Clostridiales*	*Caldicoprobacteraceae*	*Caldicoprobacter*	10	0.05	ND	
				Clostridiaceae	*Cellulosibacter*	6	0.03	44	0.24
					Clostridium sensu stricto	348	1.85	48	0.26
					Clostridium cluster III	288	1.53	909	4.83
					Tepidimicrobium	126	0.67	94	0.50
				Defluviitaleaceae	*Defluviitalea*	42	0.22	21	0.11
				Symbiobacteriaceae	*Symbiobacterium*	16	0.09	3	0.01
				Syntrophomonadaceae	*Syntrophomonas*	12	0.06	1	< 0.01
					Dethiobacter	12	0.06	3	0.02
			Halanaerobiales	*Halanaerobiaceae*	*Halothermothrix*	32	0.17	2	0.01
			Halanaerobiales	*Halanaerobiaceae*	*Halocella*	660	3.51	342	1.82
			Thermoanaerobacterales	*Thermoanaerobacteraceae*	*Tepidanaerobacter*	78	0.41	214	1.14
				Family III *incertae sedis*	*Syntrophaceticus*	31	0.16	2	0.01
		Tissierellia	*Tissierellales*	*Tissierellaceae*	*Tissierella*	4	0.02	11	0.06
				Peptoniphilaceae	*Peptoniphilus*	ND		10	0.06
	Proteobacteria	*Gammaproteobacteria*	*Alteromonadales*		*Atribacteria*[3]	10	0.05	ND	
	Spirochaetes	*Spirochaetia*	*Spirochaetales*	*Spirochaetaceae*	*Sphaerochaeta*	11	0.06	2	0.01
	Synergistetes	*Synergistia*	*Synergistales*	*Synergistaceae*	*Anaerobaculum*	33	0.18	158	0.84
	Thermotogae	*Thermotogae*	*Petrotogales*	*Petrotogaceae*	*Defluviitoga*	1040	5.53	1734	9.21

[1] In total, 18817 classified 16S rRNA gene sequences

[2] In total, 532381 classified 16S rRNA sequences

[3] *genera incertae sedis*

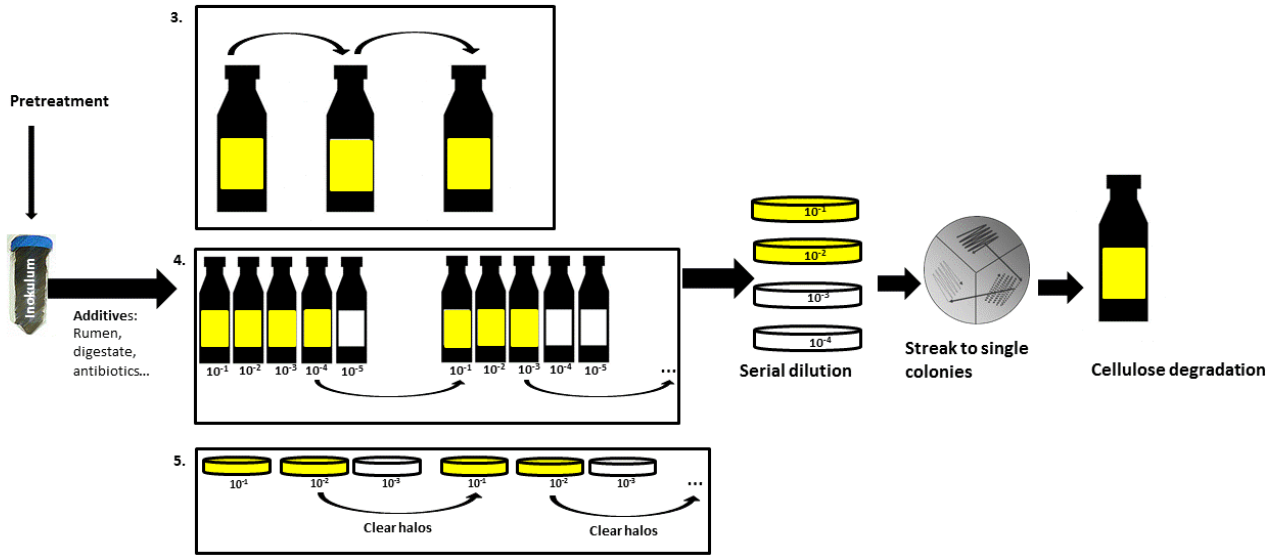

Figure S1: Exemplary work-flow illustration of the different strategies for the isolation of cellulolytic bacteria. Isolation strategy (3): direct enrichment and isolation using different media. Isolation strategy (4): serial dilution (*i.e.* dilution to extinction) in liquid medium. Isolation strategy (5): direct plating on solid medium without previous enrichment in liquid culture.

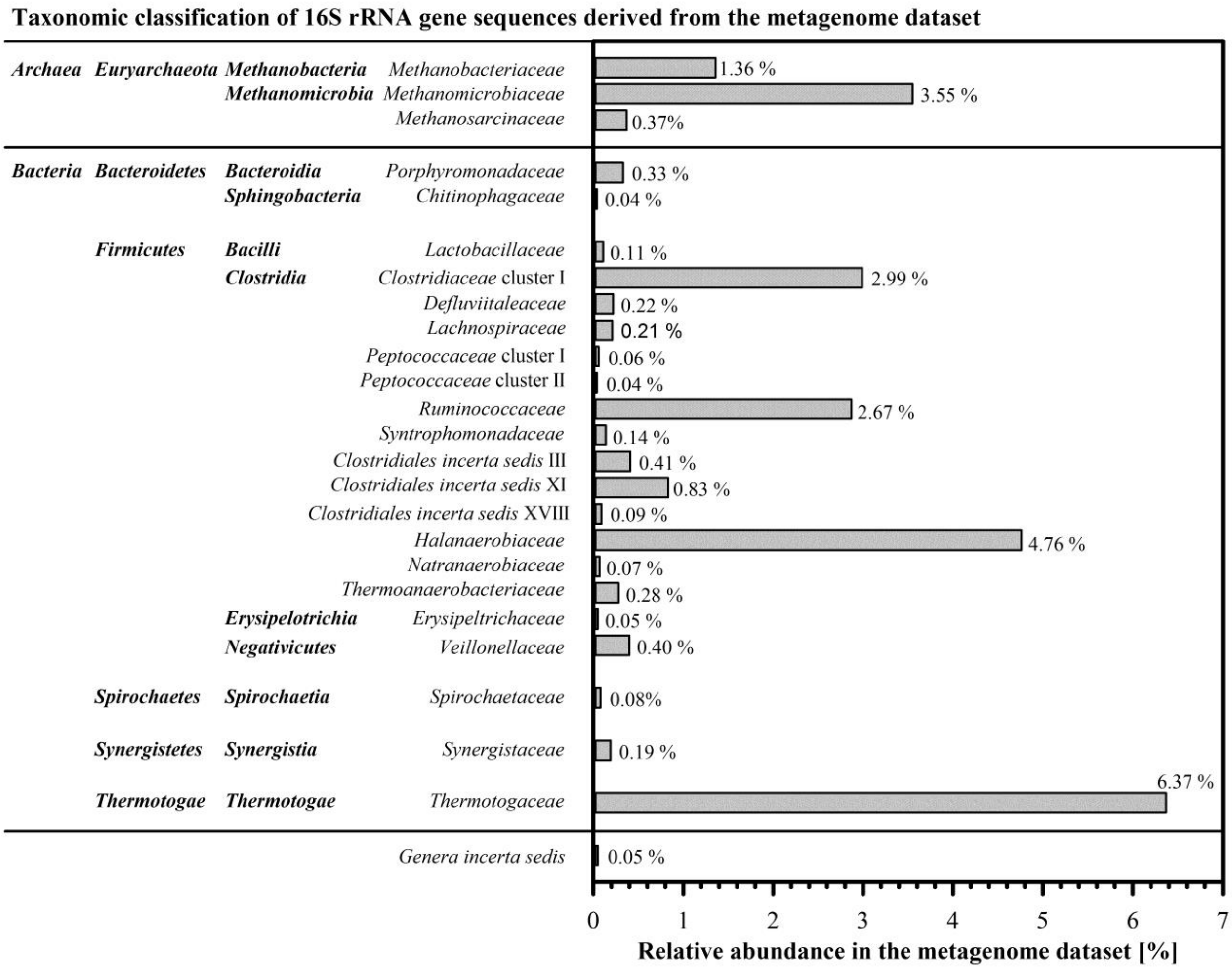

Figure S2: Relative abundance of the 25 most abundant families within the biogas microbial community of the thermophilic biogas plant as deduced from 16S rRNA gene sequences of combined metagenome datasets (>= 0.05 % of the sequences in the metagenome). Taxonomic assignments of the 16S rRNA gene sequences were obtained applying BLASTN against the RDP database applying MGX.

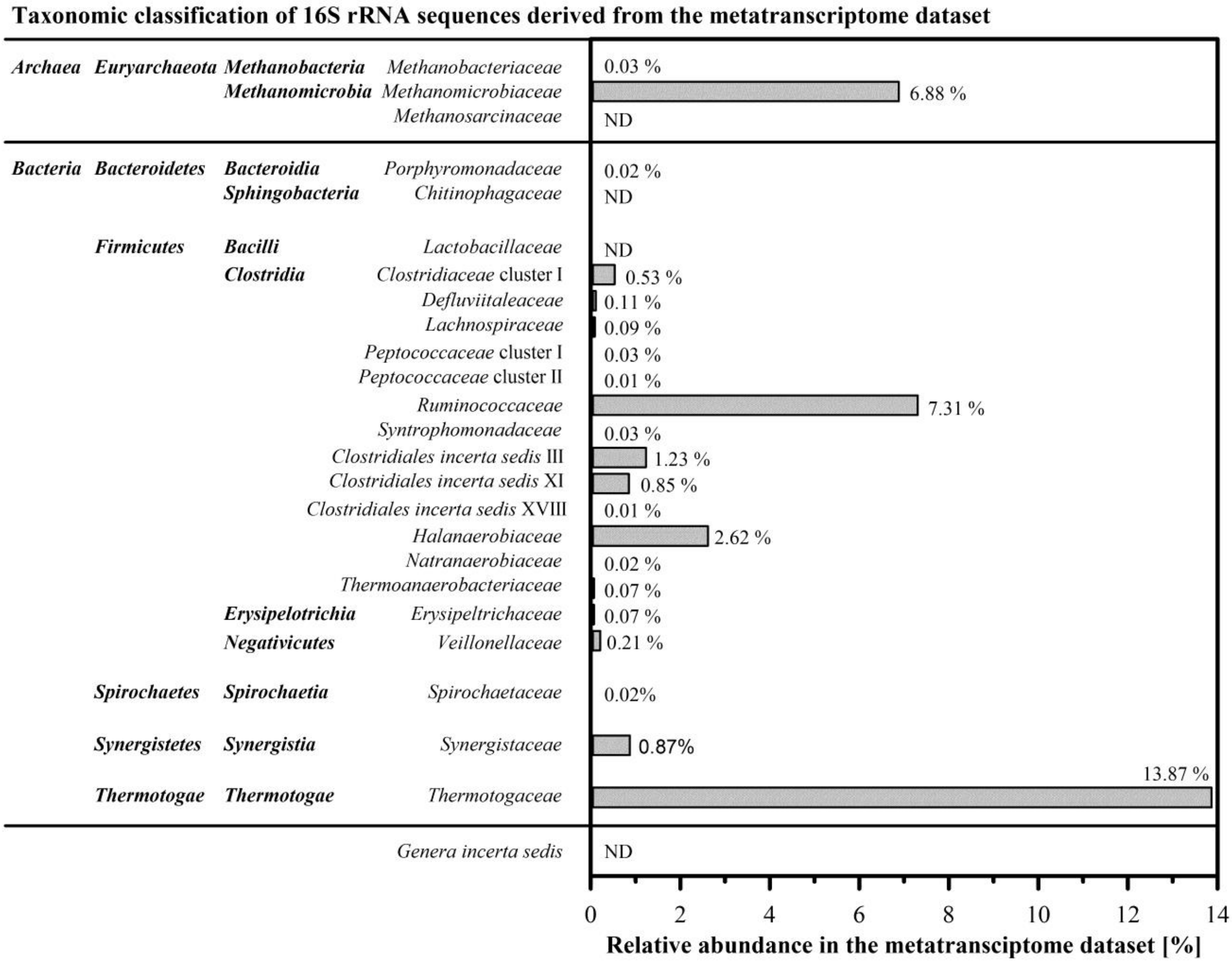

Figure S3: Relative abundance of the 25 most abundant families within the biogas microbial community of the thermophilic biogas plant as deduced from 16S rRNA sequences of the metatranscriptome datasets (>= 0.05 % of the sequences in the metatranscriptome). Taxonomic assignments of the 16S rRNA sequences were obtained applying BLASTN against the RDP database applying MGX.

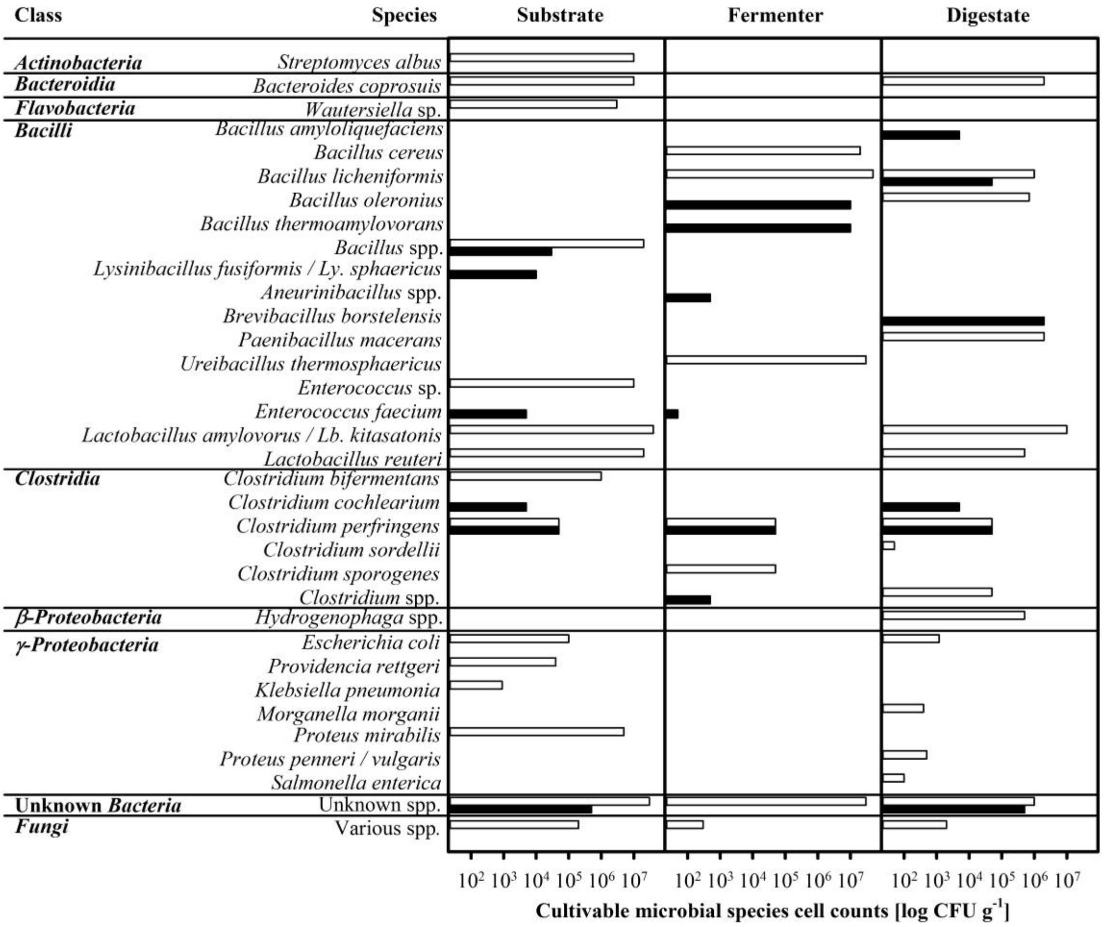

Figure S4: Cultivable microbial species applying the isolation strategies (1) - (2) and corresponding cell counts in samples of substrate (*i.e.* swine manure), fermenter content (*i.e.* main fermenter), and digestate (*i.e.* second fermenter). The cultivation temperature was 37 °C (white bars) or 50 °C (black bars), respectively.

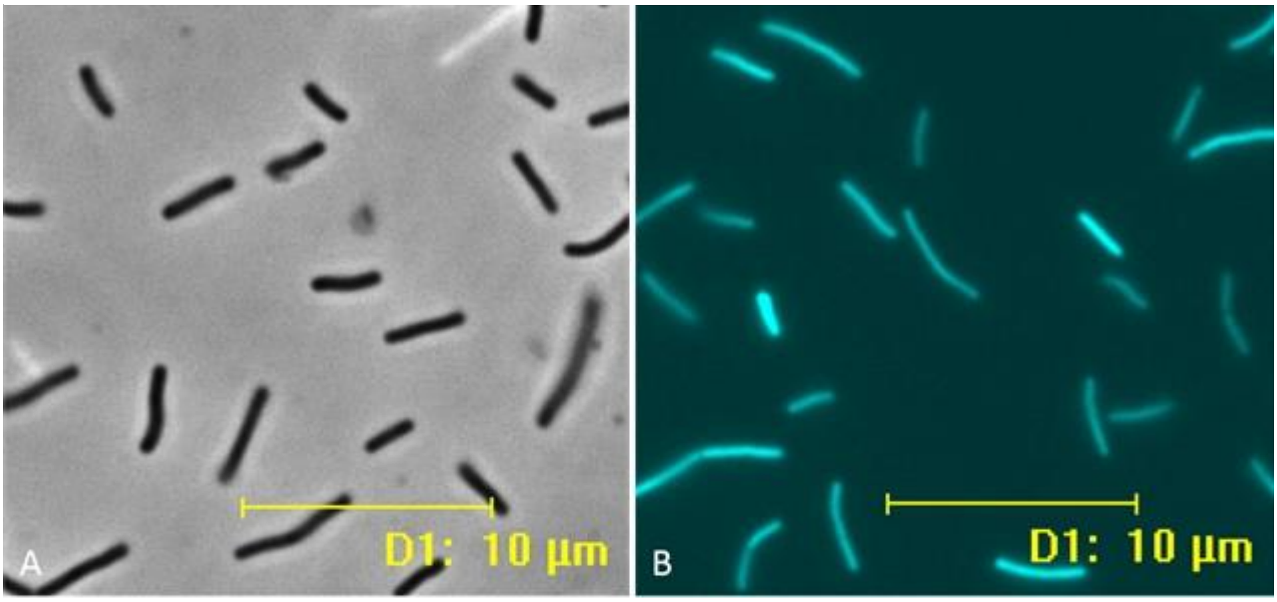

Supplementary Figure S5: Light- (a) and fluorescent (b) microscopic picture of *Methanothermobacter marburgensis* isolated from the thermophilic biogas plant.

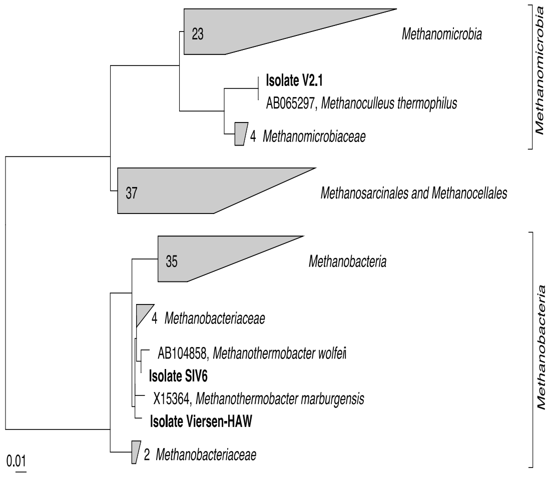

(a)

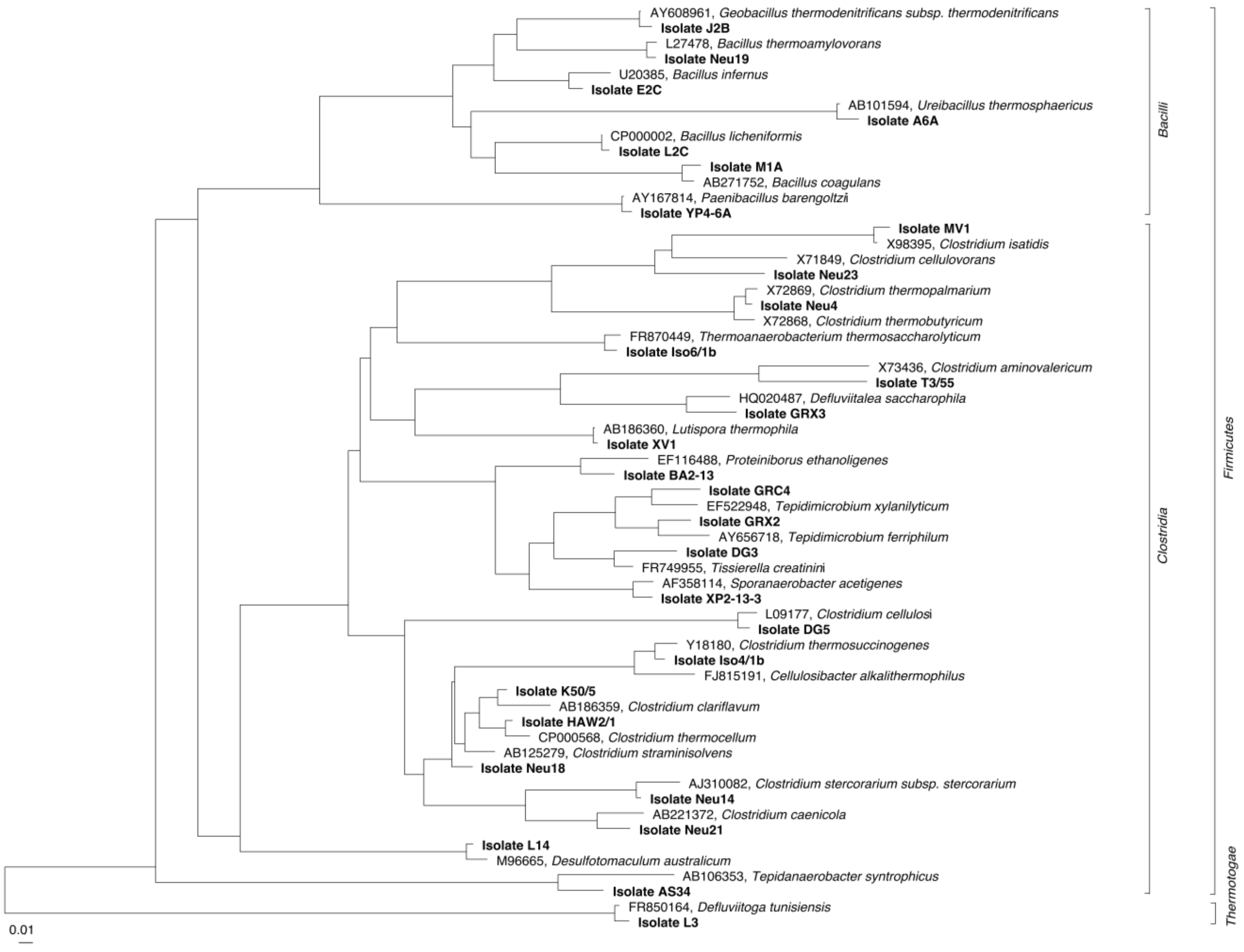

(b)

Figure S6: Phylogenetic tree of selected archaeal (a) and bacterial (b) isolates in relation to corresponding type species. The 16S rRNA gene sequences from closely related type species were obtained from the SILVA database [45]. The isolate T3/55^T represents the type strain for a novel genus and species, namely *Herbinix hemicellulosilytica*, which was recently described by [33].

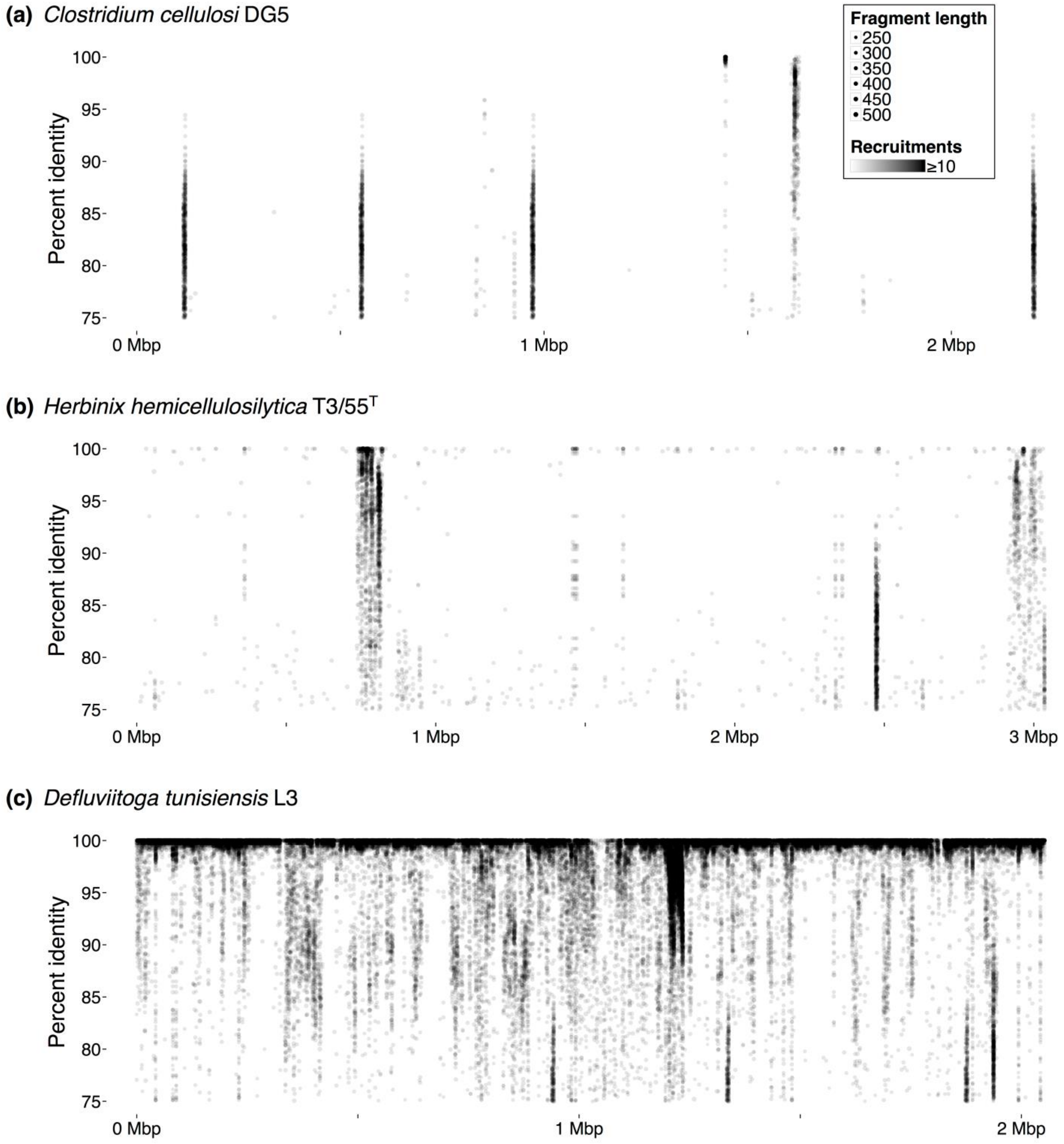

Figure S7: Fragment recruitment of metagenome sequences derived from the microbial community of the analyzed thermophilic biogas plant to the genomes of the strains *Clostridium cellulosi* str. DG5 (a), *Herbinix hemicellulosilytica* str. T3/55^{T} (b) and *Defluviitoga tunisiensis* str. L3 (c). Only metagenome sequence reads with more than 75 % sequence identity (y-axis) to the corresponding genome were mapped. Percent identity (y-axis) of a mapped metagenome read was plotted against the mapping position on the genome sequence (x-axis) of *H. hemicellulosilytica* str. T3/55^{T} or *D. tunisiensis* str. L3. The x-axis represents the extension (scale) of the *C. cellulosi* str. DG5, *H. hemicellulosilytica* str. T3/55^{T} or *D. tunisiensis* str. L3 genome. Sequence coverage is visualized by gray scale intensity (right margin). Matching sequence read length is indicated by the diameter of the circle representing the mapping position (scale is given on the top right).
